# Genetic liability to human serum metabolites is causally linked to telomere length: insights from genome-wide Mendelian randomization and metabolic pathways analysis

**DOI:** 10.3389/fnut.2024.1458442

**Published:** 2024-08-26

**Authors:** Jingwen Liu, Renbing Pan

**Affiliations:** ^1^Department of Psychiatry, Longyou People’s Hospital Affiliated with Sir Run Run Shaw Hospital, Zhejiang University School of Medicine, Quzhou, China; ^2^Department of Urology, The Quzhou Affiliated Hospital of Wenzhou Medical University, Quzhou People's Hospital, Quzhou, China

**Keywords:** serum metabolites, telomere length, metabolic pathways, Mendelian randomization, genetic liability

## Abstract

**Background:**

Telomere has been recognized as a biomarker of accelerating aging, and telomere length (TL) shortening is closely related to diverse chronic illnesses. Human serum metabolites have demonstrated close correlations with TL maintenance or shortening in observational studies. Nevertheless, little is known about the underlying pathological mechanisms, and Mendelian randomization (MR) analysis of serum metabolites may provide a more comprehensive understanding of the potential biological process.

**Methods:**

We employed a two-sample MR analysis method to assess the causal links between 486 serum metabolites and TL. We applied the inverse-variance weighted (IVW) approach as our primary analysis, and to assure the stability and robustness of our results, additional analysis methods including the weighted median, MR-Egger, and weighted mode were conducted. MR-Egger intercept test was utilized to detect the pleiotropy. Cochran’s Q test was implemented to quantify the extent of heterogeneity. Furthermore, the pathway analysis was conducted to identify potential metabolic pathways.

**Results:**

We identified 11 known blood metabolites associated with TL. Among these metabolites, four were lipid (taurocholate, dodecanedioate, 5,8-tetradecadienoate, and 15-methylpalmitate), one amino acid (levulinate (4-oxovaleate)), one carbohydrate (lactate), one nucleotide (pseudouridine), one energy (phosphate), and three xenobiotics (2-hydroxyacetaminophen sulfate, paraxanthine, and ergothioneine). The known protective metabolites included levulinate (4-oxovaleate), dodecanedioate, 5,8-tetradecadienoate, lactate, phosphate, paraxanthine, and ergothioneine. Multiple metabolic pathways have been identified as being implicated in the maintenance of telomere length.

**Conclusion:**

Our MR analysis provided suggestive evidence supporting the causal relationships between 11 identified blood metabolites and TL, necessitating further exploration to clarify the mechanisms by which these serum metabolites and metabolic pathways may affect the progression of telomeres.

## 1 Introduction

Telomeres are particular nucleoprotein structures consisting of repetitive TTAGGG sequences located at the terminal of linear chromosomes (1). On account of the unique biological character in sustaining chromosomal stability and integrity, preventing coalescence of chromosomal ends, and dictating cell proliferative history (2), telomeres have been widely acknowledged as a reliable biomarker for evaluating survival, stress, and senility (3–5). Telomere length (TL) is linked to diverse aging-related disorders, such as diabetes mellitus (6), cancer (7), cardiovascular diseases (8), Alzheimer’s disease (9), and obesity (10).

Previous research has indicated that the acceleration of TL shortening is mainly attributed to oxidative stress, immune responses, and metabolism factors, which promote cell renewal and facilitate the replication of senescent cells (11–15). Currently, accumulating evidence has indicated that various serum metabolites might be associated with telomere biological processes. Previous research performed by Nie et al. showed that maternal urinary specific polycyclic aromatic hydrocarbon (PAH) metabolite was negatively associated with cord blood TL and neonatal neurobehavioral development (16). Another study performed by Pusceddu et al. suggested a functional correlation between one-carbon metabolism and TL. In addition, they reported the availability of nucleotides and methylation groups seems to impact TL (17). Interestingly, a meta-analysis demonstrated that obesity accelerated leukocyte TL shortening in apparently healthy adults (18). Moreover, another study showed that metabolic syndrome traits, such as overweight and obesity, might facilitate the development of aging-related degenerative disorders through accelerating telomere shortening (19). Obesity stimulates oxidative stress, encompassing the production of reactive oxygen species (ROS), O_2_, and H_2_O_2_, causing telomere DNA damage and oxidation of telomerase, which subsequently results in a loss of duplication capability and accelerates the decrease of TL. In contrast, Niu et al. reported a positive association between nicotinamide mononucleotide (NMN) and TL maintenance, suggesting the administration of oral NMN supplementation during the pre-aging phase may potentially serve as an effective strategy for delaying the aging process (20). Taken together, concerning the predisposition of observational studies to underlying control bias and reversed causality (21), further investigation into the potential causal link between genetically determined human blood metabolites and TL is still needed.

Mendelian randomization (MR) is a novel epidemiology approach that leverages genetic effects to assess the inference of causality between exposures and outcomes (22). This method utilizes random genes as mediation instruments. The evidence of causal correlations provided by the MR research is similar to randomized controlled trials (RCTs) (23). The random assignment of genotypes during gamete fusion in MR analysis decreases the underlying influence of confounding factors and reversed causality (24). A previous meta-analysis of a genome-wide association study (GWAS) was performed to investigate the genetic basis for 486 human blood metabolites (25), offering an opportunity to assess the potential causality of some diseases associated with metabolic factors. For example, a previous MR study showed that genetically elevated levels of specific blood metabolites exhibited causal effects on the risk of polycystic ovary syndrome (PCOS) (26). Additionally, another MR study showed that targeted interventions of specific serum metabolites or gut microbiota could mitigate the risk of heart failure (27). Thus, by conducting a two-sample MR analysis, we could systematically evaluate the underlying causal links between serum metabolites and TL.

In this study, we aimed to employ a large-scale genetic association study to comprehensively analyze the underlying causal associations between genetically determined serum metabolite levels and TL, and to assess pooled metabolic pathways. Our findings will provide important implications for a better understanding of the association between serum metabolites and the development of telomeres. Elucidating these causal associations will provide greater insights and explore the biological mechanism of TL maintenance or shortening.

## 2 Materials and methods

### 2.1 Study design

**Figure 1:**
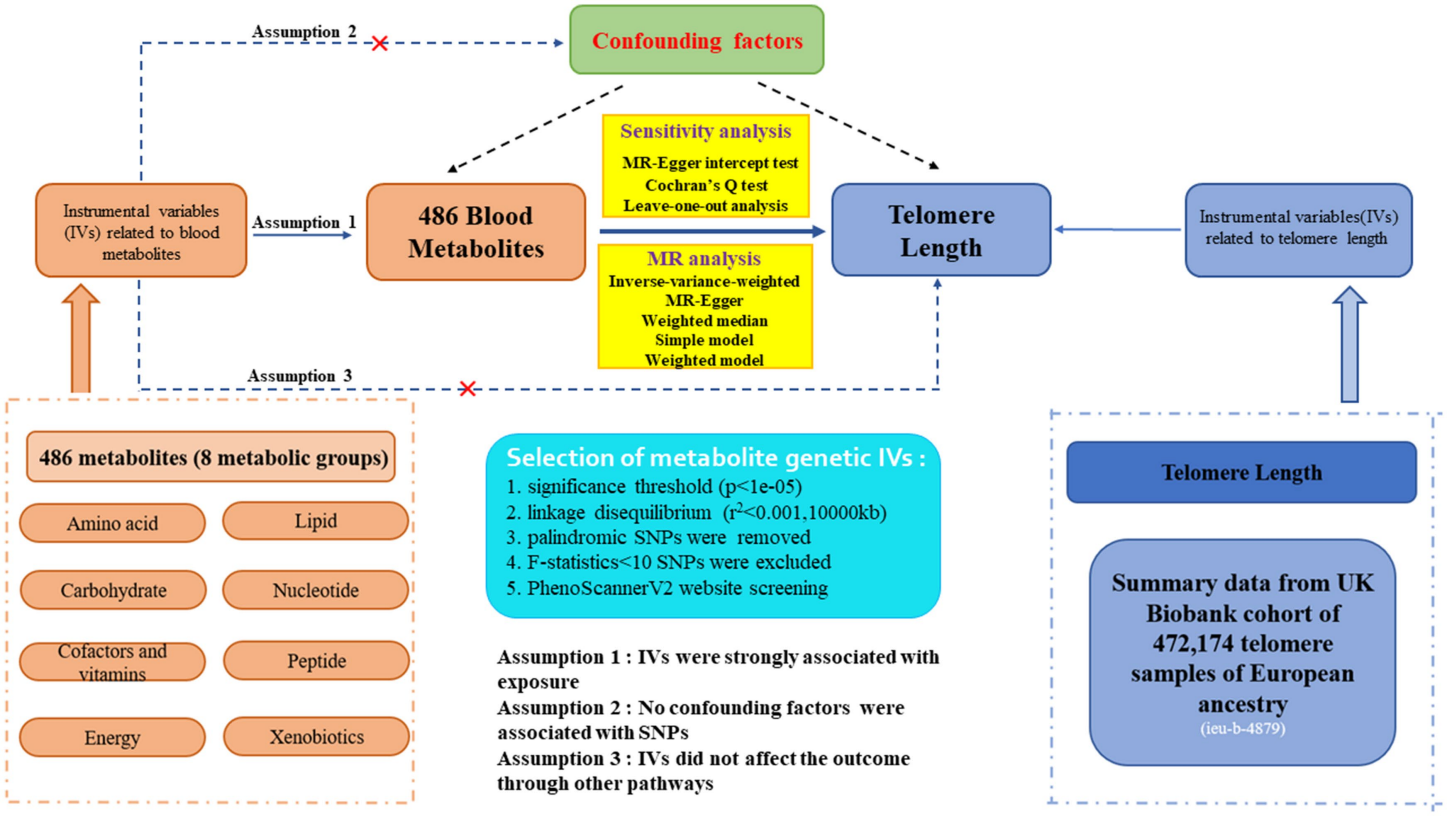
Study design, assumption and explicit information of two-sample Mendelian randomization for 486 blood metabolites and telomere length.

The flowchart of our study is displayed in Figure 1. We conducted two-sample MR analyses to assess the causal links between 486 human serum metabolites and TL by employing GWAS summary statistics, with detailed characteristics and information displayed in Supplementary Table S1. The detailed steps included the following three steps: (1) Eligible SNPs associated with 486 blood metabolites were extracted according to the specified threshold criteria. (2) The two-sample Mendelian randomization method was utilized to analyze the relationships between serum metabolites and telomere length one by one. (3) Sensitivity analysis was performed on the results of MR estimates. Additionally, to improve the reliability and robustness of the MR analysis results, instrumental variables were extracted to fulfill the following three assumptions. Firstly, the instrumental variables must be strongly linked to human blood metabolites. Secondly, no confounders are associated with the instrumental variables. Thirdly, the instrumental variables influence the outcome only through exposure and there are no other causal pathways for the instrumental variables to affect the outcome (28). We extracted genetic instrumental variables for each blood metabolite to detect the causality between each human blood metabolite and TL. The application of publicly accessible GWAS summary datasets obviated ethical approval. Our MR analysis and sensitivity analysis were performed with the R packages “Two Sample MR” and “MR-PRESSO” (version 4.2.3).

### 2.2 Data sources

#### 2.2.1 Human blood metabolite sample

The metabolite datasets involved in this study were obtained from a genome-wide association study conducted by Shin et al. (25). The blood metabolites in the whole genome-wide association study (GWAS) data were acquired from the Metabolomics GWAS server.^1^ The study comprised a total of 7,824 individuals of European ancestry and approximately 2.1 million SNPs, encompassing 1768 individuals from the KORA F4 study in Germany and 6,056 from the UK Twin Study. The unknown status was assigned to 177 out of the 486 metabolites. In addition, of the 486 metabolites, 309 metabolites were chemically classified and assigned to eight broad metabolic groups, encompassing amino acids, peptides, lipids, cofactors and vitamins, carbohydrates, energy, nucleotides, and xenobiotic metabolism. These classifications are defined by the Kyoto Encyclopedia of Genes and Genomes (KEGG) database (29). The explicit information on 486 blood metabolites is displayed in Supplementary Table S2.

#### 2.2.2 Telomere length sample

The genetically related datasets for telomere length were extracted from the publicly available GWAS (30), which was conducted by using 488,400 DNA samples from UK Biobank (UKB) individuals. The mean TL was determined using a reliable quantitative PCR assay and underwent thorough quality inspection and technical adjustments. Eventually, 472,174 telomere length measurements were retained for our MR analyses. The participants in the UK Biobank study were exclusively populations aged between 40 and 69 years, with a parallel percentage of men (45.8%) and women (54.2%).

### 2.3 Selection criteria for genetic instrumental variables (IVs)

Qualified genetic instruments related to metabolites were extracted through several rigorous control steps. Firstly, due to the lack of IVs reaching genome-wide significance, we widen the cutoff to *p*-value<1e-05 to select eligible instrumental variables, consistent with Yin et al.’s study (31). Eventually, all 486 blood metabolites were identified utilizing this standard. Secondly, Linkage disequilibrium (LD) clumping was performed on the candidate instrumental variables to identify independent ones (*r*^2^ < 0.001 with 10,000 kb), assuring independence among SNPs for each exposure. Thirdly, intermediate allele frequency palindromic SNPs were removed resulting from allele frequencies that were not provided in the GWAS of blood metabolites. Fourthly, to avoid bias resulting from weak instrumental variables, we figured out the F-statistics for each SNP to estimate statistical strength (32, 33). SNPs with F-statistics greater than 10 were regarded as robust instrumental variables for our study (34). Additionally, the outcome-related SNPs (*p*-value<1e-05) were removed. Moreover, to fulfill the independent assumption of MR, SNPs associated with confounding factors, encompassing blood pressure, body mass index (BMI), and smoking, were removed by PhenoScannerV2 website screening. The brief extraction criteria of serum metabolites genetic IVs in our study are demonstrated in Figure 1.

### 2.4 MR preliminary analysis

To detect the causal links of human blood metabolites on TL by connecting different SNPs, we performed a two-sample Mendelian randomization analysis by utilizing several common analytical models. We applied standard IVW estimates as the dominant analysis, which combined the Wald ratio estimation of each SNP, manifesting the highest statistical power among different MR methods (35). This method has been acknowledged as the principal approach for assessing the underlying causal associations between human blood metabolites and TL. Our analysis needed data on SNPs, alleles, effect sizes, *p*-values, and allele frequencies (EAF) (36). Furthermore, additional MR analysis methods, such as MR-Egger, weighted median, simple mode, and weighted mode were implemented as auxiliary analysis methods to IVW by augmenting more robust estimates in a wider range of situations. The MR-Egger approach can discover the violations of the instrumental variables assumption and provide consistent estimates with invalid instruments (37). We employed complementary analyses of MR methods with various modeling assumptions and merits (weighted median and weighted mode) to enhance the reliability and validity of our findings.

### 2.5 Sensitivity analyses

To validate the exclusive influence of IVs on the outcome through exposure and enhance the robustness of the findings, sensitivity analysis is also required. Thus, multiple approaches encompassing weighted median, MR-Egger regression, Cochran’s Q statistics, and leave-one-out (LOO) analysis were applied to verify the reliability and validity of the significant results (P_IVW_ < 0.05). Among them, the weighted median approach enhances causal effect detection and eliminates type I errors. The MR-Egger intercept test was conducted to explore the presence of horizontal pleiotropy, with statistical significance defined as *p*-values for the intercept being less than 0.05 (38). Additionally, Cochran’s Q statistics were utilized for IVW and MR-Egger to estimate heterogeneity among instrumental variables. A p-value greater than 0.05 in Cochran’s Q statistics suggested the absence of heterogeneity among IVs (39). Finally, we applied LOO analysis to determine whether the findings were impacted by a single SNP (40). Additionally, we also conducted the MR Steiger directionality test to ensure whether our results supported our hypothesis (41). Lastly, we adopted a multiple-testing-adjusted threshold of *p* < 1.03e-04 (0.05/486) utilizing the Bonferroni correction to declare a statistically significant causality (42). We also reported metabolites that had a *p* < 0.05, but were above the Bonferroni-corrected criteria, as suggestive association with TL. Therefore, potential eligible candidate metabolites for participation in TL development were identified by multiple standards: (1) *p*-value for the dominant MR analysis was significant (P_IVW_ < 0.05). (2) Consistent direction and magnitude of the additional approaches with the IVW method results. (3) No heterogeneity or horizontal pleiotropy was detected. (4) MR estimates are not severely influenced by a single SNP in LOO analysis.

### 2.6 Metabolic pathway analysis

The chosen metabolite metabolic pathway analysis was investigated by utilizing the web-based Metaconflict 5.0^2^ (43). Functional enrichment analyses and the metabolic pathway module were employed to identify potential metabolite categories or pathways that may be associated with the biological process of TL development. The metabolite databases, including the Small Molecule Pathway Database (SMPDB) and the Kyoto Encyclopedia of Genes and Genomes (KEGG) database, were utilized in this study. Notably, only metabolites exceeding the advised threshold were analyzed for metabolic pathways (P_IVW_ < 0.05).

## 3 Results

### 3.1 Selection of the instrumental variables

We conducted two-sample MR analyses to assess the causal links of genetically determined metabolites on TL. Considering the restricted genetic variance, as well as the limited number of SNPs and low statistic powers, the MR analysis was performed by widening the threshold to *p*-value<1e-05. The number of candidate SNPs for 486 metabolites ranged from 3 to 478, with a median number of 15 (Supplementary Table S2). Additionally, the minimum F-statistics of these instrumental variables was 17.64, suggesting that all SNPs were sufficiently effective for the MR analysis and that the weak instrumental bias was impossible to occur (all F-statistics >10).

### 3.2 Causality of genetically determined metabolites on telomere length

**Figure 2:**
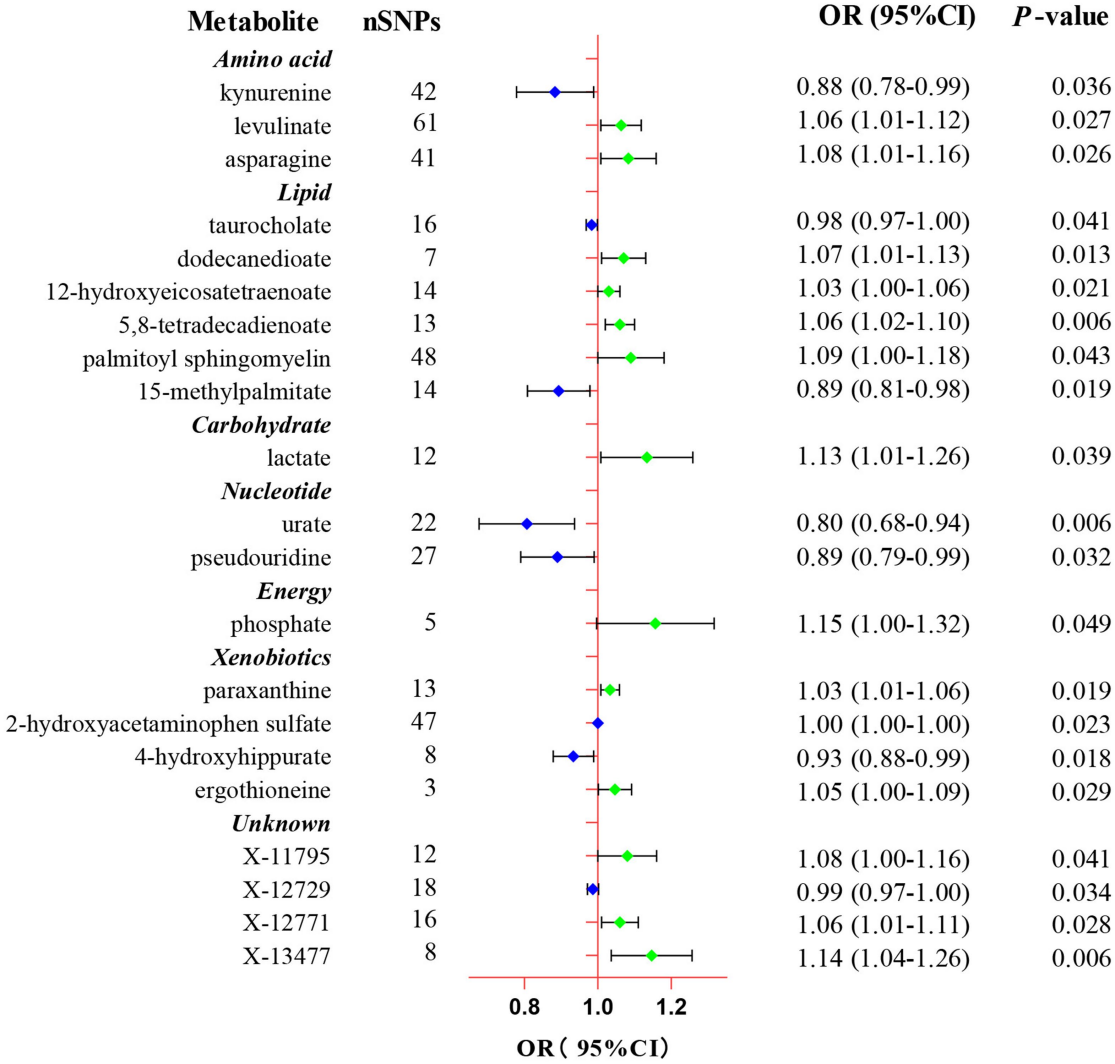
Forest plot of the Mendelian randomization analysis for the associations between 21 eligible candidate metabolites and telomere length. CI, confidence interval; OR, odds ratio; SNP, single nucleotide polymorphism.

**Figure 3:**
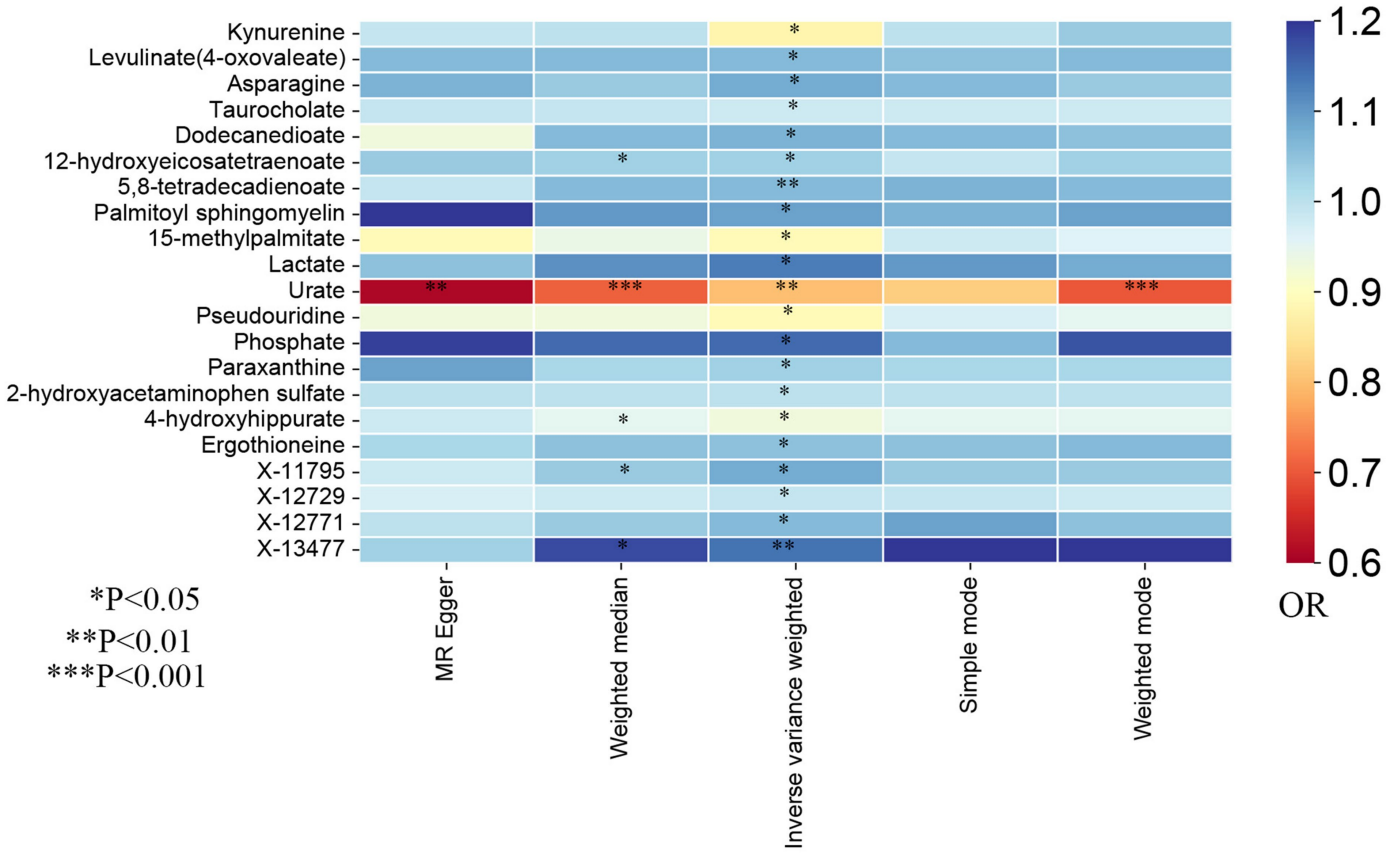
The heatmaps of five Mendelian randomization analysis methods. Different color blocks represent different odds ratio values. OR, odds ratio.

In the present study, the IVW model was used as the primary method in evaluating the causal associations between 486 human blood metabolites and TL. Although no significant causal links of blood metabolites with telomere length were exhibited by utilizing the Bonferroni correction, a total of 21 blood metabolites comprising 17 known metabolites and 4 unknown metabolites demonstrated suggestive associations with TL at the standard significance level of 0.05 (P_IVW_ < 0.05, Figure 2). As shown in Figure 2, the 17 known metabolites were chemically allocated to the amino acids, lipids, carbohydrates, energy, nucleotides, and xenobiotics. Furthermore, the MR-Egger, weighted mode, simple mode, and weighted median methods yield consistent causal estimates in terms of both direction and magnitude. To visually represent the strength of instrumental variables and make the compiled data more intuitive, we created heatmaps to exhibit this part of the data (Figure 3). The detailed MR estimates of diverse approaches are presented in Supplementary Table S3.

### 3.3 Sensitivity analysis

Because IVW approaches are susceptible to weak instrumental bias, sensitivity analyses were performed to ensure the reliability and stability of the causality. Table 1 shows the sensitivity analysis results for evaluating the robustness of our MR estimates. MR-Egger intercept test manifested no significant pleiotropy except urate (P_Egger-intercept_ = 0.045). Furthermore, MR-Egger and IVW Cochran’s Q statistics were applied to investigate the heterogeneity. The absence of substantial heterogeneity was evident among SNPs for those identified metabolites except kynurenine, asparagine, 12-hydroxyeicosatetraenoate, palmitoyl sphingomyelin, urate, 4-hydroxyhippurate, and X-12771 (Q-P_IVW_ and Q-P_MR-Egger_ as shown in Table 1).

**Table 1:** Heterogeneity and horizontal pleiotropy analysis for the 21 metabolites identified by the IVW method.

Metabolites	Subcategory	Pleiotropy		Heterogeneity			
		MR-Egger intercept test		MR-Egger		IVW	
		Intercept	*P-*value	Q-statistic	*P-*value	Q-statistic	*P-*value
Kynurenine	Amino acid	−0.0016	0.305	170.33	5.15e-18	174.93	1.80e-18
Levulinate (4-oxovaleate)	Amino acid	0.000012	0.985	62.36	0.358	62.36	0.392
Asparagine	Amino acid	0.0018	0.818	56.02	0.038	56.10	0.047
Taurocholate	Lipid	−0.0011	0.333	17.61	0.225	18.87	0.220
Dodecanedioate	Lipid	0.0048	0.226	2.59	0.768	4.46	0.615
12-hydroxyeicosatetraenoate	Lipid	−0.00096	0.733	24.13	0.019	24.37	0.028
5,8-tetradecadienoate	Lipid	0.00274	0.135	7.11	0.791	9.71	0.641
Palmitoyl sphingomyelin	Lipid	−0.0011	0.456	66.48	0.026	67.30	0.028
15-methylpalmitate	Lipid	−0.0001	0.960	20.66	0.056	20.66	0.079
Lactate	Carbohydrate	0.00099	0.713	13.82	0.182	14.01	0.232
Urate	Nucleotide	0.00406	0.045	53.79	6.22e-05	66.05	1.49e-06
Pseudouridine	Nucleotide	−0.00047	0.785	28.09	0.304	28.18	0.349
Phosphate	Energy	−0.00067	0.708	1.34	0.719	1.51	0.825
Paraxanthine	Xenobiotics	−0.0029	0.247	10.91	0.451	12.40	0.414
2-hydroxyacetaminophen sulfate	Xenobiotics	−0.00028	0.805	48.88	0.320	48.95	0.356
4-hydroxyhippurate	Xenobiotics	−0.0019	0.740	14.37	0.026	14.66	0.041
Ergothioneine	Xenobiotics	0.0036	0.818	0.20	0.657	0.28	0.868
X-11795	Unknown	0.0023	0.368	9.58	0.478	10.47	0.489
X-12729	Unknown	0.0015	0.299	13.07	0.668	14.22	0.651
X-12771	Unknown	0.0018	0.401	24.46	0.040	25.77	0.041
X-13477	Unknown	0.0023	0.381	5.77	0.449	6.67	0.464

Eventually, after combining complementary methods and sensitivity analyses, 11 known metabolites that met the rigorous screening standard were identified as significantly eligible candidates. Specifically, taurocholate (OR: 0.98, 95%CI: 0.97–1.00, P_IVW_ = 0.041), 15-methylpalmitate (OR: 0.89, 95%CI: 0.81–0.98, P_IVW_ = 0.019), pseudouridine (OR: 0.89, 95%CI: 0.79–0.99, P_IVW_ = 0.032), and 2-hydroxyacetaminophen sulfate (OR: 1.00, 95%CI: 1.00–1.00, P_IVW_ = 0.023) were the most notably risky metabolites for TL. In contrast, levulinate (4-oxovaleate) (OR: 1.06, 95%CI: 1.01–1.12, P_IVW_ = 0.027), dodecanedioate (OR: 1.07, 95%CI: 1.01–1.13, P_IVW_ = 0.013), 5,8-tetradecadienoate (OR: 1.06, 95%CI: 1.01–1.10, P_IVW_ = 0.006), lactate (OR: 1.13, 95%CI: 1.01–1.26, P_IVW_ = 0.039), phosphate (OR: 1.15, 95%CI: 1.00–1.32, P_IVW_ = 0.049), paraxanthine (OR: 1.03, 95%CI: 1.01–1.06, P_IVW_ = 0.019), and ergothioneine (OR: 1.05, 95%CI: 1.00–1.09, P_IVW_ = 0.029) were factors with highest protective value for TL. In addition, the forest plots of other analysis methods for these 11 metabolites were showed in Supplementary Figure S1. Furthermore, of these eligible candidates, 5,8-tetradecadienoate (P_IVW_ = 0.006) showed the most significant causal relationship with telomere length.

**Figure 4:**
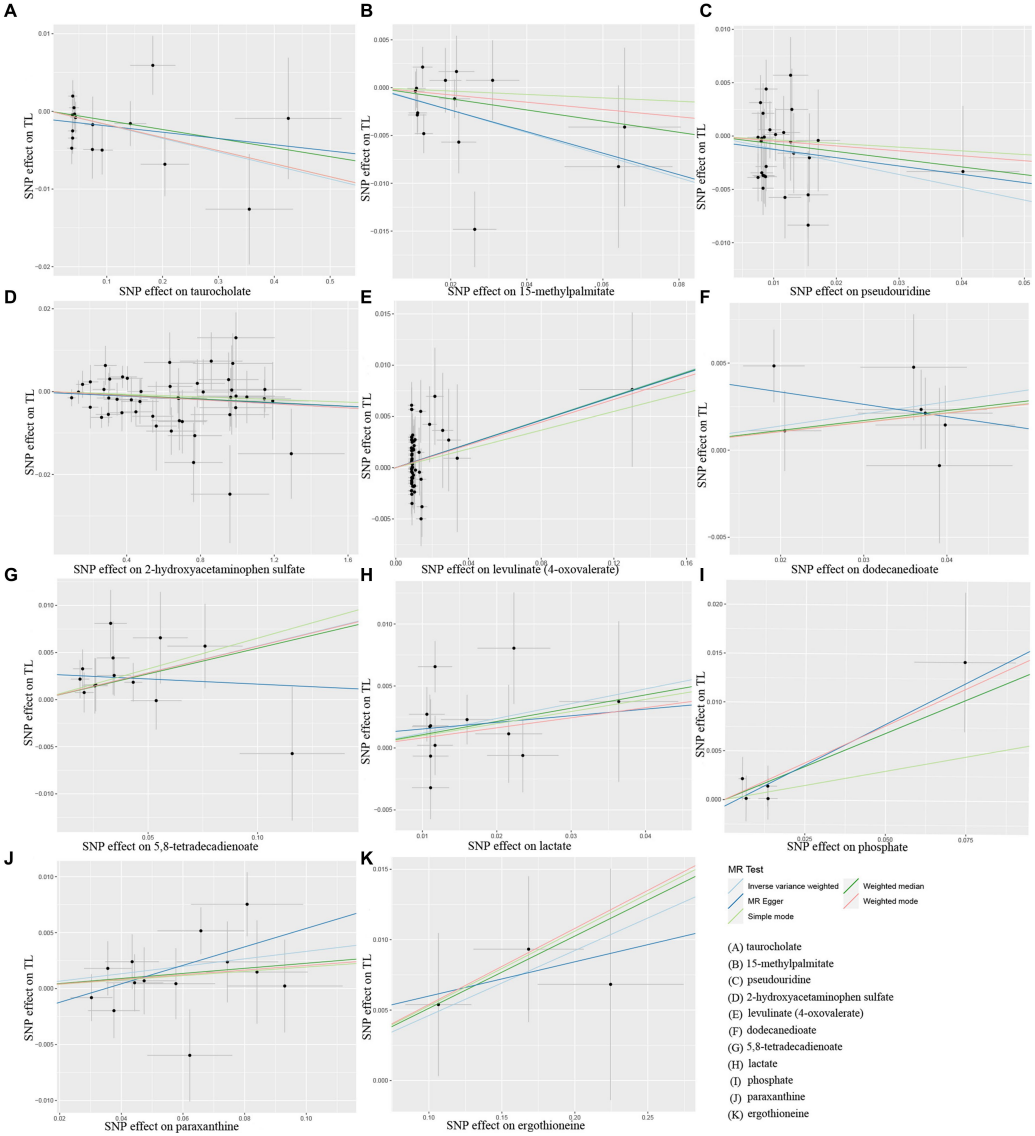
Scatter plots of Mendelian randomization (MR) analyses between 11 identified metabolites and telomere length (TL). SNP, single nucleotide polymorphism. **(A)** taurocholate, **(B)** 15-methylpalmitate, **(C)** pseudouridine, **(D)** 2-hydroxyacetaminophen sulfate, **(E)** levulinate (4-oxovaleate), **(F)** dodecanedioate, **(G)** 5,8-tetradecadienoate, **(H)** lactate, **(I)** phosphate, **(J)** paraxanthine, **(K)** ergothioneine.

**Figure 5:**
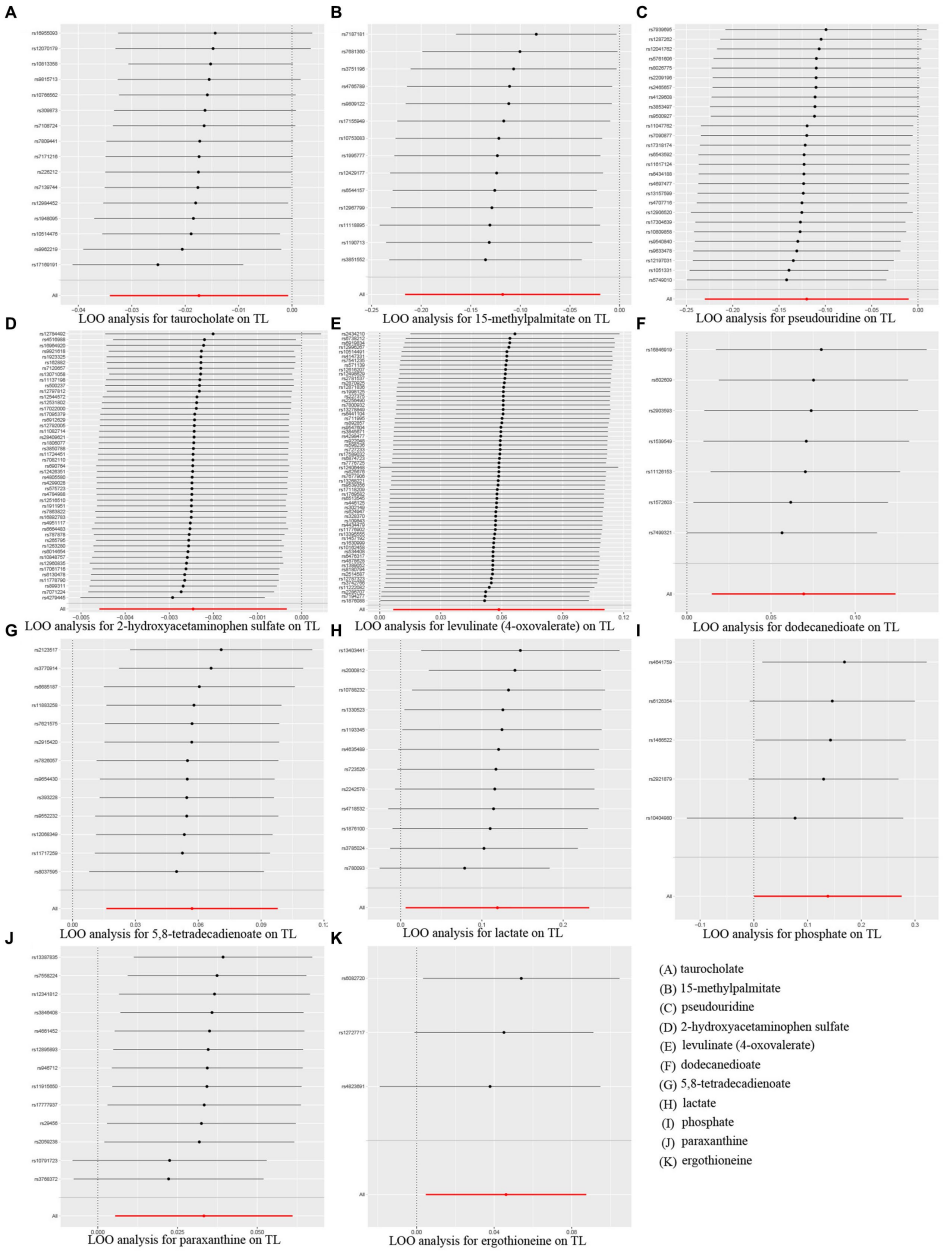
MR leave-one-out (LOO) sensitivity analysis to assess whether every single SNP drove the causal association of 11 identified metabolites on telomere length (TL). **(A)** taurocholate, **(B)** 15-methylpalmitate, **(C)** pseudouridine, **(D)** 2-hydroxyacetaminophen sulfate, **(E)** levulinate (4-oxovaleate), **(F)** dodecanedioate, **(G)** 5,8-tetradecadienoate, **(H)** lactate, **(I)** phosphate, **(J)** paraxanthine, **(K)** ergothioneine.

Additionally, scatter plots of associations of 11 genetically determined metabolites with TL were shown in Figure 4. The findings of the LOO analysis similarly suggested that none of the single SNPs exerted significant influence on the analysis outcomes (Figure 5). Moreover, the distribution of SNPs was shown in the funnel plots (Supplementary Figure S2). Finally, the results demonstrated that the *p*-values of 11 identified metabolites were all between 1.03e-04 and 0.05, indicating that these metabolites were suggestively linked to the development of TL. Further studies are needed to validate their associations in the future.

### 3.4 Confounding analysis and identification of significant genes

**Figure 6:**
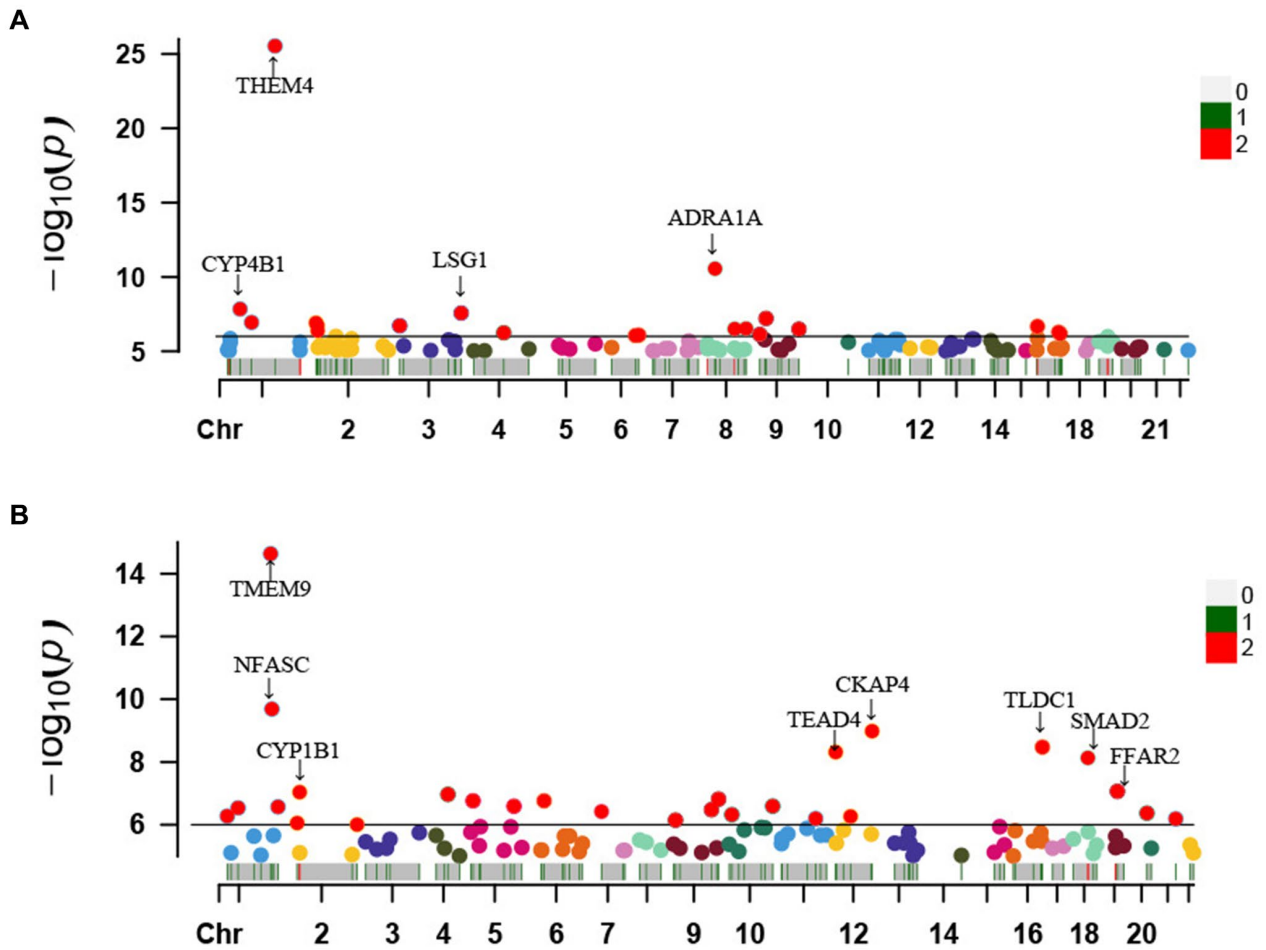
Manhattan plots exhibited the distribution of genetic locus associated with 7 protective metabolites **(A)** and 4 risky metabolites **(B)**. The significant genes were labelled. Chr, chromosome.

For these seven protective metabolites and four risky metabolites, we further manually detected metabolism-related instrumental variables for the second most common traits (smoking, blood pressure, BMI, and diabetes). Looking closely at the PhenoScannerV2 online platform, we found that 218 metabolite-related SNPs were not associated with confounding factors. The corresponding gene information of instrumental variables is shown in Supplementary Table S4. Furthermore, Manhattan plots exhibited the distribution of genetic locus associated with seven protective metabolites (Figure 6A) and four risky metabolites (Figure 6B). Significant genes were labeled in Manhattan plots and the significantly protective genes for telomere length maintenance were THEM4, ADRA1A, LSG1, and CYP4B1.

### 3.5 Metabolic pathways analysis

**Figure 7:**
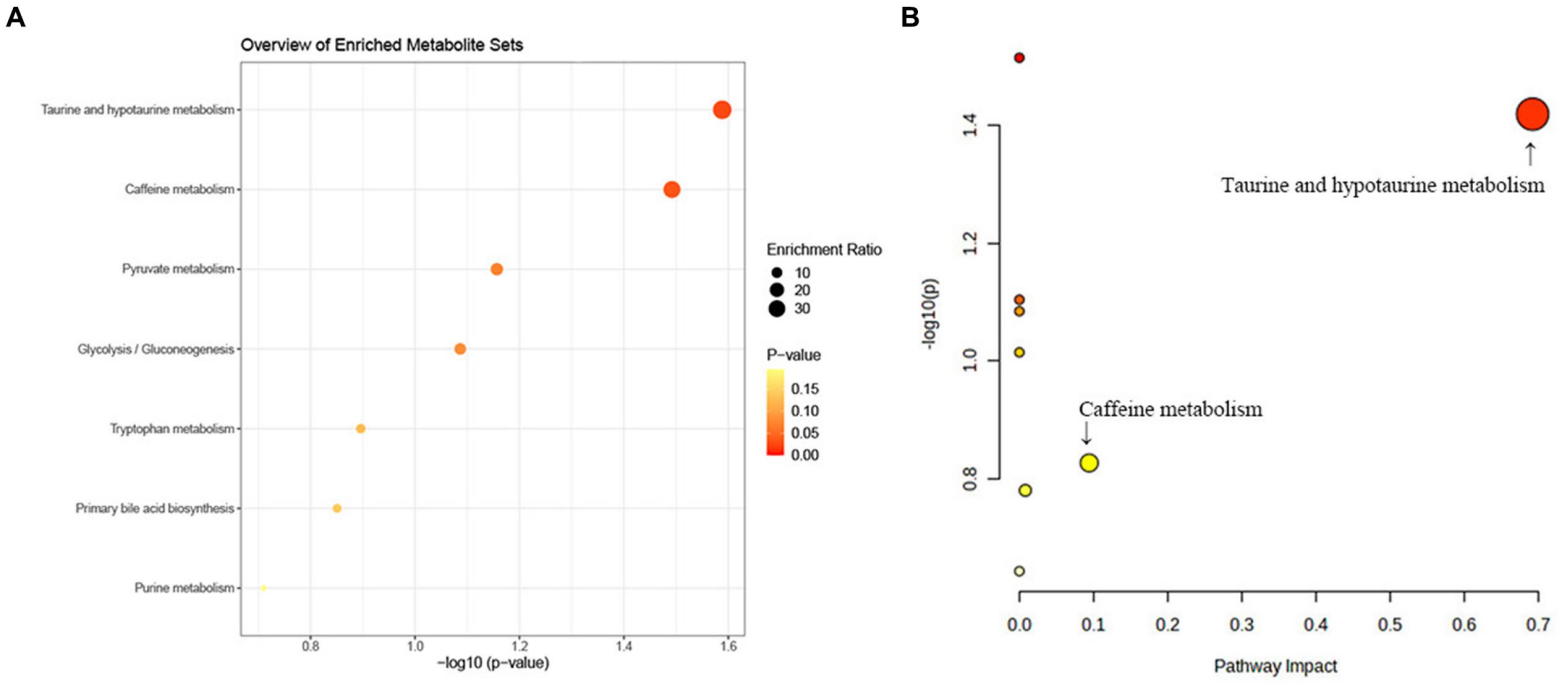
KEGG pathway functional enrichment analysis **(A)** and metabolic pathway **(B)** analysis of identified metabolites by MetaboAnalyst5.0.

We further carried out the metabolic pathway analysis utilizing all metabolites identified through the IVW approach (P_IVW_ < 0.05). The results of functional enrichment analyses and the metabolic pathway analysis are demonstrated in Figure 7. Notably, this study identified two significant metabolic pathways that were involved in the biological process of telomeres development (Supplementary Table S5). The significant metabolite taurocholate was involved in the taurine and hypotaurine metabolism pathway (*p* < 0.05).

## 4 Discussion

In this study, we elucidated the causal links between 486 genetically predicted serum metabolites and TL by employing genetic variation as instrumental variables in a two-sample Mendelian randomization model. Through rigorous screening standards and extensive sensitivity analysis, 11 known metabolites were identified as the significantly eligible candidates, which were causally associated with TL. We discovered that four metabolites (taurocholate, 15-methylpalmitate, pseudouridine, and 2-hydroxyacetaminophen sulfate) belonging to the lipid, nucleotide, and xenobiotic super pathways may increase the risk of TL shortening. In contrast, seven metabolites (levulinate, dodecanedioate, 5,8-tetradecadienoate, lactate, phosphate, paraxanthine, and ergothioneine) belonging to the amino acid, lipid, carbohydrate, energy, and xenobiotics super pathways were protective factors for TL maintenance. Additionally, multiple metabolic pathways, encompassing taurine and hypotaurine metabolism and caffeine metabolism, were involved in the biological process related to telomeres development.

Our study identified four lipids (taurocholate, dodecanedioate, 5,8-tetradecadienoate, and 15-methylpalmitate) causally associated with the TL maintaining or shortening. Coincidentally, a previous experimental study conducted by Guo et al. showed that TL is inherited maternally, and the reduction in TL among Chinese participants may be attributed to impaired lipid metabolism, which aligns with our findings (44). Moreover, previous metabolomics research has unequivocally suggested that lipid metabolism plays a crucial role in TL maintenance. Diverse metabolites derived from fatty acids, including glycerophosphocholine, lysophospholipids, glycerides, and phosphatidlcholine, were closely related to TL (45). In particular, our findings demonstrated that 5,8-tetradecadienoate (OR: 1.06, 95%CI: 1.02–1.10, P_IVW_ = 0.006) was the most significant protective factor for TL maintenance in the four lipids. These findings underscore the complex correlation between lipid metabolism and TL, providing a robust foundation for further investigation in this field. However, further investigation to detect the accurate biological mechanism for lipids on TL was required in the future.

The taurine and hypotaurine metabolism pathway and caffeine metabolism pathway were identified to be associated with the telomeres biological process by metabolite pathway analysis. We discovered that taurocholate, which participated in the taurine and hypotaurine metabolic pathway, may play a crucial role in the telomere development process (metabolism pathway analysis: *p*-value = 0.031). These results indicated that taurocholate may serve as a potential therapeutic target in TL maintenance and delaying senescence. Nevertheless, the epidemiological evidence for the association between taurocholate and TL is limited. Thus, further clinical and experimental studies were warranted to elucidate the mechanisms between taurocholate and TL shortening. In addition, our research demonstrated that the caffeine metabolism pathway may be involved in the biological process of telomeres. Coincidentally, previous observational studies manifested that instant coffee intake had a causal effect on TL shortening (2), suggesting caffeine may play a pivotal role in TL shortening.

Additionally, the causal links between multiple serum metabolites and TL were evaluated utilizing MR analysis in this study. Consistent with our findings, previous observational studies in the literature revealed that a high concentration of serum urate was negatively associated with TL (46). TL shortening was attributed to oxidative damage and other post-processing events in both proliferating and non-proliferating cells (4). The presence of urate induces cellular stress and signaling events, including the generation of reactive oxygen species (ROS) and activation of inflammatory signaling pathways, leading to telomeres shortening. In addition, the production of ROS occurs concomitantly with the intracellular urate formation catalyzed by xanthine oxidases (47). The augment of xanthine oxidase activity facilitates intracellular urate generation. Meanwhile, the increased extracellular urate concentration exacerbates the flux of urate into cells. The occurrence of these events results in the intracellular accumulation of urate, which exacerbates the production of ROS and apoptosis (48). Eventually, these cellular events result in TL shortening, in turn, TL shortening further aggravates cell stress, forming a vicious spiral (49). These mechanisms indicated that serum urate decreasing may be beneficial to control telomere shortening and delaying cellular senescence. In our study, despite the results of sensitivity analysis showing heterogeneity and pleiotropy, the causal effects of urate on TL shortening were highly significant (OR: 0.80, 95%CI: 0.68–0.94, P_IVW_ = 0.006). These sensitivity analysis variations could be ascribed to the different options of instrumental variables and GWAS data.

Our study has several strengths. Firstly, as far as we know, this is the first MR analysis to combine metabolomics and genomics to systematically assess the causality of human blood metabolites on TL. Such a design can eliminate limitations correlated with confounders often encountered in conventional observational studies and can provide more robust evidence of causality between exposure and outcome. Secondly, utilizing the most extensive and current GWAS attainable for metabolites enabled the establishment of a robust instrumental variable, predicted to yield unbiased MR estimates. Thirdly, we conducted several sensitivity analyses (Egger intercept test and Cochran’s Q statistics), which can control the bias caused by the pleiotropic effect to ensure the validity and stability of MR findings. Fourthly, we identified eligible candidate metabolites causally associated with telomeres development by multiple standards and rigorous control steps, including heterogeneity, pleiotropy, and LOO analysis.

However, several limitations should be acknowledged in our study. First, the choice of instrumental variables was conducted utilizing a wide P threshold (*p* < 1e-05), and this might result in consequence bias and false-positive variants. Similarly, a previous study has also utilized the identical cutoff when evaluating the causal relationships between 486 blood metabolites and diabetic retinopathy (50). Second, the individuals of GWAS included in our study were restricted to European ancestry, so caution should be exercised when extrapolating these results to other populations. Third, despite performing various sensitivity analyses to verify MR assumptions, we cannot eliminate the influence of horizontal pleiotropy and reverse causality. Fourth, on account of the effects of diverse blood metabolites on the body being complicated and interactive, to satisfy the independent assumption of MR (excluding confounding factors), performing a phenome-wide association study of the instrumental variables may be useful. Lastly, our study is limited to public databases and lacks real-world clinical sample tests. Hence, these underlying links need to be validated in larger cohorts and the potential biological mechanism of genetically determined blood metabolites and metabolic pathways in the regulation of TL maintenance or shortening should be further detected in future research.

## 5 Conclusion

To sum up, we conducted a comprehensive MR study to identify 11 genetically determined human serum metabolites causally associated with telomere length, among which four were negatively associated and seven were positively associated. Compared with the other 10 metabolites, 5,8-tetradecadienoate has a significantly causal association with telomere length maintenance. Additionally, taurocholate may be involved in the metabolism biological process of telomere length maintenance via specific metabolic pathways.

## Data Availability

The original contributions presented in the study are included in the article/Supplementary material, further inquiries can be directed to the corresponding author.

## Footnotes

^1^ https://metabolomics.helmholtz-muenchen.de/gwas/

^2^ http://www.metaboanalyst.ca/

## References

[1] Blackburn EH Greider CW Szostak JW. Telomeres and telomerase: the path from maize, tetrahymena and yeast to human cancer and aging. Nat Med. (2006) 12:1133–8. doi: 10.1038/nm1006-1133, PMID: 17024208

[2] Wei Y Li Z Lai H Lu P Zhang B Song L. Instant coffee is negatively associated with telomere length: finding from observational and Mendelian randomization analyses of UK biobank. Nutrients. (2023) 15, 1–15. doi: 10.3390/nu15061354, PMID: 36986083 PMC10055626

[3] Turner KJ Vasu V Griffin DK. Telomere biology and human phenotype. Cells. (2019) 8, 1–19. doi: 10.3390/cells8010073 PMC6356320 30669451

[4] Lin J Epel E. Stress and telomere shortening: insights from cellular mechanisms. Ageing Res Rev. (2022) 73:101507. doi: 10.1016/j.arr.2021.101507 34736994 PMC8920518

[5] Liu J Wang L Wang Z Liu JP. Roles of telomere biology in cell senescence, replicative and chronological ageing. Cells. (2019) 8, 1–10. doi: 10.3390/cells8010054 PMC6356700 30650660

[6] Zee RY Castonguay AJ Barton NS Germer S Martin M. Mean leukocyte telomere length shortening and type 2 diabetes mellitus: a case-control study. Transl Res. (2010) 155:166–9. doi: 10.1016/j.trsl.2009.09.012, PMID: 20303464

[7] Rode L Nordestgaard BG Bojesen SE. Long telomeres and cancer risk among 95 568 individuals from the general population. Int J Epidemiol. (2016) 45:1634–43. doi: 10.1093/ije/dyw179, PMID: 27498151

[8] Sagris M Theofilis P Antonopoulos AS Tsioufis K Tousoulis D. Telomere length: a cardiovascular biomarker and a novel therapeutic target. Int J Mol Sci. (2022) 23, 1–13. doi: 10.3390/ijms232416010 PMC9781338 36555658

[9] Scheller Madrid A Rasmussen KL Rode L Frikke-Schmidt R Nordestgaard BG Bojesen SE. Observational and genetic studies of short telomeres and Alzheimer's disease in 67,000 and 152,000 individuals: a Mendelian randomization study. Eur J Epidemiol. (2020) 35:147–56. doi: 10.1007/s10654-019-00563-w, PMID: 31564046

[10] Kim S Parks CG DeRoo LA Chen H Taylor JA Cawthon RM. Obesity and weight gain in adulthood and telomere length. Cancer Epidemiol Biomarkers Prev. (2009) 18:816–20. doi: 10.1158/1055-9965.EPI-08-0935, PMID: 19273484 PMC2805851

[11] Halvorsen TL Beattie GM Lopez AD Hayek A Levine F. Accelerated telomere shortening and senescence in human pancreatic islet cells stimulated to divide in vitro. J Endocrinol. (2000) 166:103–9. doi: 10.1677/joe.0.1660103, PMID: 10856888

[12] Kurz DJ Decary S Hong Y Trivier E Akhmedov A Erusalimsky JD. Chronic oxidative stress compromises telomere integrity and accelerates the onset of senescence in human endothelial cells. J Cell Sci. (2004) 117:2417–26. doi: 10.1242/jcs.01097, PMID: 15126641

[13] von Zglinicki T. Oxidative stress shortens telomeres. Trends Biochem Sci. (2002) 27:339–44. doi: 10.1016/S0968-0004(02)02110-2 12114022

[14] Lopez-Doriga A Valle L Alonso MH Ausso S Closa A Sanjuan X. Telomere length alterations in microsatellite stable colorectal cancer and association with the immune response. Biochim Biophys Acta Mol Basis Dis. (1864) 2018:2992–3000. 10.1016/j.bbadis.2018.06.010 29908233

[15] van der Spek A Karamujic-Comic H Pool R Bot M Beekman M Garmaeva S. Fat metabolism is associated with telomere length in six population-based studies. Hum Mol Genet. (2022) 31:1159–70. doi: 10.1093/hmg/ddab281 34875050

[16] Nie J Li J Cheng L Deng Y Li Y Yan Z. Prenatal polycyclic aromatic hydrocarbons metabolites, cord blood telomere length, and neonatal neurobehavioral development. Environ Res. (2019) 174:105–13. doi: 10.1016/j.envres.2019.04.024, PMID: 31055168

[17] Pusceddu I Herrmann M Kirsch SH Werner C Hubner U Bodis M. One-carbon metabolites and telomere length in a prospective and randomized study of B-and/or D-vitamin supplementation. Eur J Nutr. (2017) 56:1887–98. doi: 10.1007/s00394-016-1231-z 27379829

[18] Khosravaniardakani S Bokov DO Mahmudiono T Hashemi SS Nikrad N Rabieemotmaen S. Obesity accelerates leukocyte telomere length shortening in apparently healthy adults: a meta-analysis. Front Nutr. (2022) 9:812846. doi: 10.3389/fnut.2022.812846, PMID: 35719148 PMC9199514

[19] Loh NY Rosoff D Noordam R Christodoulides C. Investigating the impact of metabolic syndrome traits on telomere length: a Mendelian randomization study. Obesity (Silver Spring). (2023) 31:2189–98. doi: 10.1002/oby.23810, PMID: 37415075 PMC10658743

[20] Niu KM Bao T Gao L Ru M Li Y Jiang L. The impacts of short-term NMN supplementation on serum metabolism, fecal microbiota, and telomere length in pre-aging phase. Front Nutr. (2021) 8:756243. doi: 10.3389/fnut.2021.756243, PMID: 34912838 PMC8667784

[21] Mi J Liu Z Jiang L Li M Wu X Zhao N. Mendelian randomization in blood metabolites identifies triglycerides and fatty acids saturation level as associated traits linked to pancreatitis risk. Front Nutr. (2022) 9:1021942. doi: 10.3389/fnut.2022.1021942, PMID: 36299997 PMC9589364

[22] Gao N Ni M Song J Kong M Wei D Dong A. Causal relationship between tea intake and cardiovascular diseases: a Mendelian randomization study. Front Nutr. (2022) 9:938201. doi: 10.3389/fnut.2022.938201, PMID: 36225867 PMC9548982

[23] Smith GD. Mendelian randomization for strengthening causal inference in observational studies: application to gene x environment interactions. Perspect Psychol Sci. (2010) 5:527–45. doi: 10.1177/1745691610383505 26162196

[24] Wei T Zhu Z Liu L Liu B Wu M Zhang W. Circulating levels of cytokines and risk of cardiovascular disease: a Mendelian randomization study. Front Immunol. (2023) 14:1175421. doi: 10.3389/fimmu.2023.1175421 37304261 PMC10247976

[25] Shin SY Fauman EB Petersen AK Krumsiek J Santos R Huang J. An atlas of genetic influences on human blood metabolites. Nat Genet. (2014) 46:543–50. doi: 10.1038/ng.2982, PMID: 24816252 PMC4064254

[26] Sun S Jiao M Han C Zhang Q Shi W Shi J. Causal effects of genetically determined metabolites on risk of polycystic ovary syndrome: a Mendelian randomization study. Front Endocrinol (Lausanne). (2020) 11:621. doi: 10.3389/fendo.2020.00621, PMID: 33013699 PMC7505923

[27] Luo Q Hu Y Chen X Luo Y Chen J Wang H. Effects of gut microbiota and metabolites on heart failure and its risk factors: a two-sample Mendelian randomization study. Front Nutr. (2022) 9:899746. doi: 10.3389/fnut.2022.899746, PMID: 35799593 PMC9253861

[28] Pan R Xiao M Wu Z Liu J Wan L. Associations of genetically predicted circulating levels of cytokines with telomere length: a Mendelian randomization study. Front Immunol. (2023) 14:1276257. doi: 10.3389/fimmu.2023.1276257, PMID: 37942318 PMC10628532

[29] Kanehisa M Goto S Sato Y Furumichi M Tanabe M. KEGG for integration and interpretation of large-scale molecular data sets. Nucleic Acids Res. (2012) 40:D109–14. doi: 10.1093/nar/gkr988, PMID: 22080510 PMC3245020

[30] Codd V Wang Q Allara E Musicha C Kaptoge S Stoma S. Polygenic basis and biomedical consequences of telomere length variation. Nat Genet. (2021) 53:1425–33. doi: 10.1038/s41588-021-00944-6, PMID: 34611362 PMC8492471

[31] Yin Y Shan C Han Q Chen C Wang Z Huang Z. Causal effects of human serum metabolites on occurrence and progress indicators of chronic kidney disease: a two-sample Mendelian randomization study. Front Nutr. (2023) 10:1274078. doi: 10.3389/fnut.2023.1274078 38260086 PMC10800733

[32] Burgess S Thompson SG CRP CHD Genetics Collaboration. Avoiding bias from weak instruments in Mendelian randomization studies. Int J Epidemiol. (2011) 40:755–64. doi: 10.1093/ije/dyr036, PMID: 21414999

[33] Bowden J Del Greco MF Minelli C Davey Smith G Sheehan NA Thompson JR. Assessing the suitability of summary data for two-sample Mendelian randomization analyses using MR-egger regression: the role of the I2 statistic. Int J Epidemiol. (2016) 45:1961–74. doi: 10.1093/ije/dyw220, PMID: 27616674 PMC5446088

[34] Palmer TM Lawlor DA Harbord RM Sheehan NA Tobias JH Timpson NJ. Using multiple genetic variants as instrumental variables for modifiable risk factors. Stat Methods Med Res. (2012) 21:223–42. doi: 10.1177/0962280210394459, PMID: 21216802 PMC3917707

[35] Burgess S Butterworth A Thompson SG. Mendelian randomization analysis with multiple genetic variants using summarized data. Genet Epidemiol. (2013) 37:658–65. doi: 10.1002/gepi.21758, PMID: 24114802 PMC4377079

[36] Choi KW Chen CY Stein MB Klimentidis YC Wang MJ Koenen KC. Assessment of bidirectional relationships between physical activity and depression among adults: a 2-sample Mendelian randomization study. JAMA Psychiatry. (2019) 76:399–408. doi: 10.1001/jamapsychiatry.2018.4175 30673066 PMC6450288

[37] Bowden J Davey Smith G Burgess S. Mendelian randomization with invalid instruments: effect estimation and bias detection through egger regression. Int J Epidemiol. (2015) 44:512–25. doi: 10.1093/ije/dyv080, PMID: 26050253 PMC4469799

[38] Shao F Yao Y Weng D Wang R Liu R Zhang Y. Causal association of plasma circulating metabolites with nephritis: a Mendelian randomization study. Front Nutr. (2024) 11:1364841. doi: 10.3389/fnut.2024.1364841, PMID: 38765814 PMC11099270

[39] Burgess S Bowden J Fall T Ingelsson E Thompson SG. Sensitivity analyses for robust causal inference from Mendelian randomization analyses with multiple genetic variants. Epidemiology. (2017) 28:30–42. doi: 10.1097/EDE.0000000000000559, PMID: 27749700 PMC5133381

[40] Burgess S Thompson SG. Interpreting findings from Mendelian randomization using the MR-egger method. Eur J Epidemiol. (2017) 32:377–89. doi: 10.1007/s10654-017-0255-x 28527048 PMC5506233

[41] Zhang X Zhou J Xie Z Li X Hu J He H. Exploring blood metabolites and thyroid disorders: a bidirectional Mendelian randomization study. Front Endocrinol (Lausanne). (2023) 14:1270336. doi: 10.3389/fendo.2023.1270336, PMID: 37876541 PMC10591305

[42] Curtin F Schulz P. Multiple correlations and Bonferroni's correction. Biol Psychiatry. (1998) 44:775–7. doi: 10.1016/S0006-3223(98)00043-2 9798082

[43] Pang Z Chong J Zhou G de Lima Morais DA Chang L Barrette M. MetaboAnalyst 5.0: narrowing the gap between raw spectra and functional insights. Nucleic Acids Res. (2021) 49:W388–96. doi: 10.1093/nar/gkab382, PMID: 34019663 PMC8265181

[44] Guo L Chen Y Li H Yin F Ge M Hu L. Telomere length is maternally inherited and associated with lipid metabolism in Chinese population. Aging (Albany NY). (2022) 14:354–67. doi: 10.18632/aging.203810, PMID: 34995210 PMC8791204

[45] Zhao J Zhu Y Uppal K Tran VT Yu T Lin J. Metabolic profiles of biological aging in American Indians: the strong heart family study. Aging (Albany NY). (2014) 6:176–86. doi: 10.18632/aging.100644 PMID: 24799415 PMC4012935

[46] Lv Z Cui J Zhang J. Associations between serum urate and telomere length and inflammation markers: evidence from UK biobank cohort. Front Immunol. (2022) 13:1065739. doi: 10.3389/fimmu.2022.1065739, PMID: 36591268 PMC9797991

[47] Kimura Y Tsukui D Kono H. Uric acid in inflammation and the pathogenesis of atherosclerosis. Int J Mol Sci. (2021) 22, 1–19. doi: 10.3390/ijms222212394, PMID: 34830282 PMC8624633

[48] Shen S He F Cheng C Xu B Sheng J. Uric acid aggravates myocardial ischemia-reperfusion injury via ROS/NLRP3 pyroptosis pathway. Biomed Pharmacother. (2021) 133:110990. doi: 10.1016/j.biopha.2020.110990, PMID: 33232925

[49] Barnes RP Fouquerel E Opresko PL. The impact of oxidative DNA damage and stress on telomere homeostasis. Mech Ageing Dev. (2019) 177:37–45. doi: 10.1016/j.mad.2018.03.013, PMID: 29604323 PMC6162185

[50] Yang C Ma Y Yao M Jiang Q Xue J. Causal relationships between blood metabolites and diabetic retinopathy: a two-sample Mendelian randomization study. Front Endocrinol (Lausanne). (2024) 15:1383035. doi: 10.3389/fendo.2024.1383035 38752182 PMC11094203

