# Supplementary material for: Genetic liability to human serum metabolites is causally linked to telomere length: insights from genome-wide Mendelian randomization and metabolic pathways analysis
Source: Front Nutr. 2024 Aug 26;11:1458442. doi: 10.3389/fnut.2024.1458442 (PMC11381963; doi:10.3389/fnut.2024.1458442)

**Figure S1.** The forest plots of other analysis approaches for eleven metabolites on telomere length.


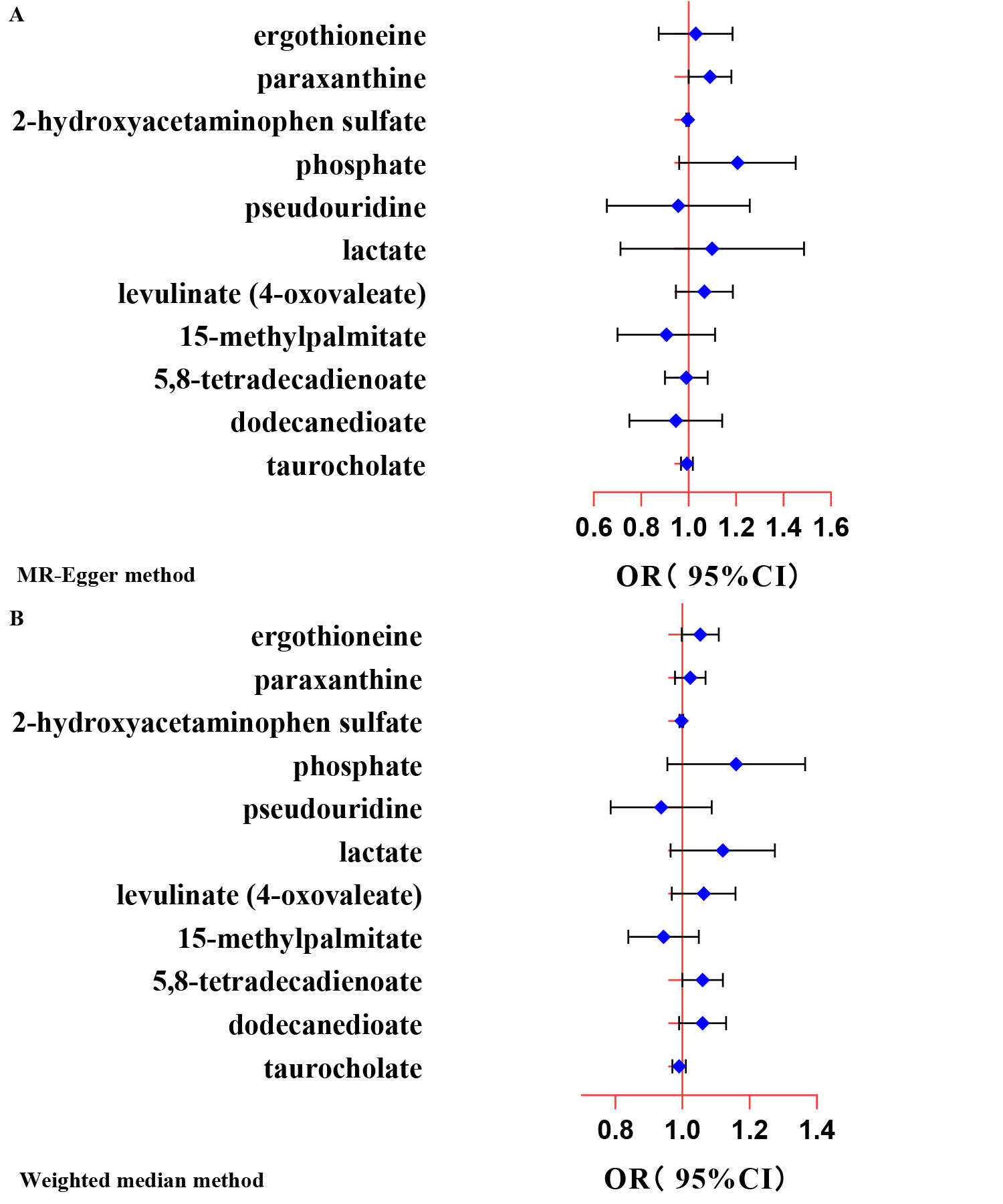

Supplement: Supplementary file 2 [file Data_Sheet_1.ZIP › Supplementary materials/Supplementary Figure S1.docx]
