# Supplementary material for: Genetic liability to human serum metabolites is causally linked to telomere length: insights from genome-wide Mendelian randomization and metabolic pathways analysis
Source: Front Nutr. 2024 Aug 26;11:1458442. doi: 10.3389/fnut.2024.1458442 (PMC11381963; doi:10.3389/fnut.2024.1458442)

**Figure S2.** The funnel plots demonstrated the distribution of diverse metabolites instrumental variables.


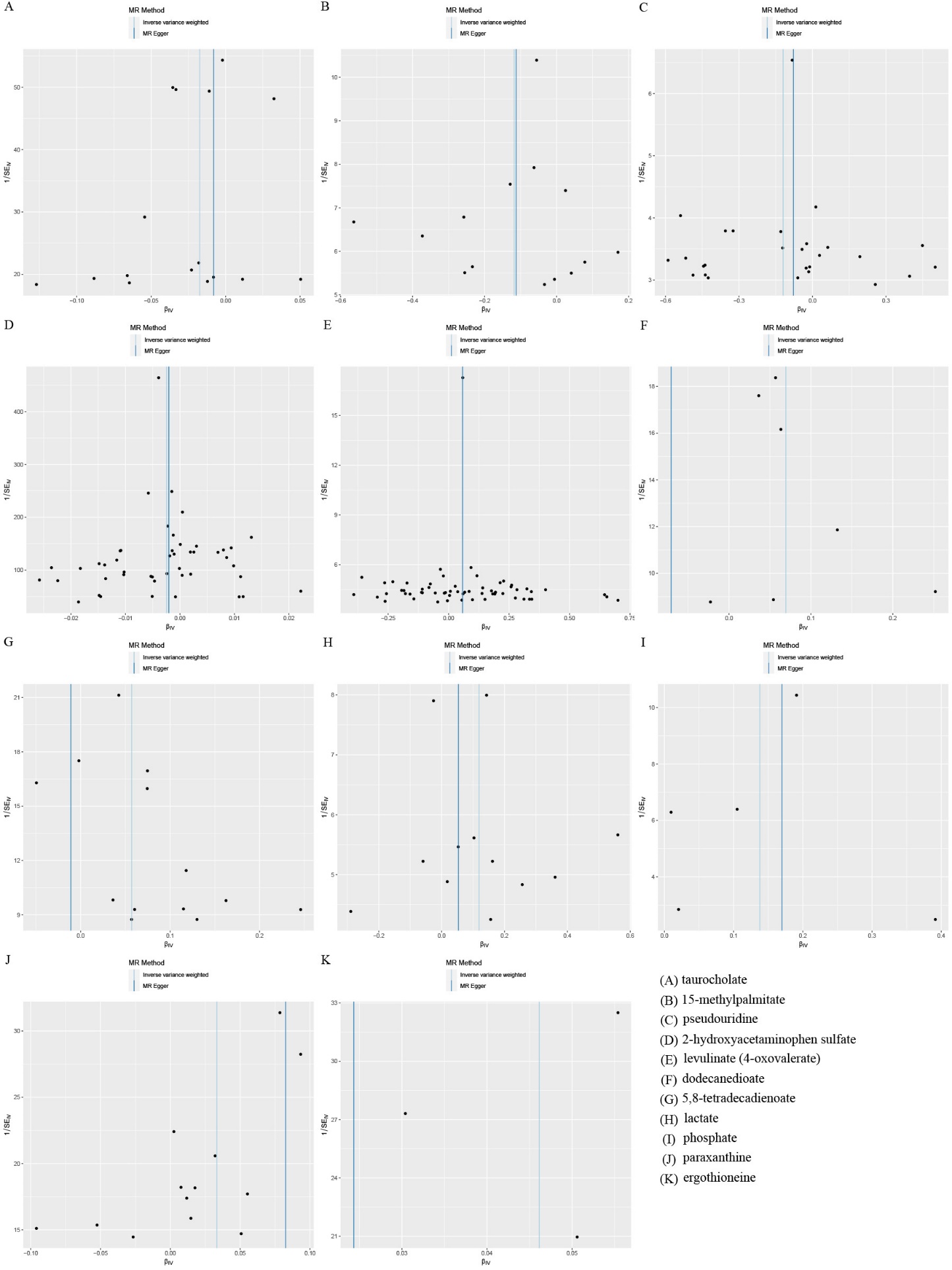

Supplement: Supplementary file 2 [file Data_Sheet_1.ZIP › Supplementary materials/Supplementary Figure S2.docx]
