# Supplementary material for: Genetic liability to human serum metabolites is causally linked to telomere length: insights from genome-wide Mendelian randomization and metabolic pathways analysis
Source: Front Nutr. 2024 Aug 26;11:1458442. doi: 10.3389/fnut.2024.1458442 (PMC11381963; doi:10.3389/fnut.2024.1458442)
Supplement: Supplementary file 2 [file Data_Sheet_1.ZIP › Supplementary materials/Supplementary Table S1.docx]

**Table S1.** Brief characteristics description of 486 human blood metabolites and telomere length GWAS cohorts involved in this study.

| **Exposure or outcome** | **Source** | **Sample size** | **Ancestry** | **Access Link** | **PMID** |
| --- | --- | --- | --- | --- | --- |
| 486 human blood metabolites | KORA F4 and UK Twin study | 7,824 participants | European | http://www.ncbi.nlm.nih.gov/pubmed/24816252 | 24816252 |
| Telomere length | UK Biobank | 472,174 samples | European | http://gwas.mrcieu.ac.uk/datasets/ieu-b-4879/ | 34611362 |
