# Supplementary material for: Genetic liability to human serum metabolites is causally linked to telomere length: insights from genome-wide Mendelian randomization and metabolic pathways analysis
Source: Front Nutr. 2024 Aug 26;11:1458442. doi: 10.3389/fnut.2024.1458442 (PMC11381963; doi:10.3389/fnut.2024.1458442)
Supplement: Supplementary file 2 [file Data_Sheet_1.ZIP › Supplementary materials/Supplementary Table S3.docx]

**Table S3.** MR analysis of the associations between 21 eligible candidate metabolites and telomere length.

| **Candidate human blood metabolites** | | **Number of SNPs** | **OR (95% CI)** | **P-value** | **P for Cochran’s Q test** | **P for Egger intercept test** |
| --- | --- | --- | --- | --- | --- | --- |
| **Kynurenine** | |  |  |  |  |  |
|  | MR Egger | 42 | 0.99 (0.77 - 1.26) | 0.929 | 5.15e-18 | 0.305 |
|  | Weighted median | 42 | 1.00 (0.92 - 1.10) | 0.934 |  |  |
|  | Inverse variance weighted | 42 | 0.88 (0.78- 0.99) | 0.036 | 1.80e-18 |  |
|  | Simple mode | 42 | 1.00 (0.85 - 1.17) | 0.966 |  |  |
|  | Weighted mode | 42 | 1.04 (0.92- 1.17) | 0.509 |  |  |
| **Levulinate (4-oxovaleate)** | |  |  |  |  |  |
|  | MR Egger | 61 | 1.06 (0.95 - 1.19) | 0.319 | 0.358 | 0.985 |
|  | Weighted median | 61 | 1.06 (0.97- 1.16) | 0.224 |  |  |
|  | Inverse variance weighted | 61 | 1.06 (1.01- 1.12) | 0.027 | 0.392 |  |
|  | Simple mode | 61 | 1.05 (0.88- 1.25) | 0.608 |  |  |
|  | Weighted mode | 61 | 1.06 (0.95- 1.17) | 0.305 |  |  |
| **Asparagine** | |  |  |  |  |  |
|  | MR Egger | 41 | 1.07 (0.94 - 1.21) | 0.323 | 0.038 | 0.818 |
|  | Weighted median | 41 | 1.04 (0.95 - 1.14) | 0.387 |  |  |
|  | Inverse variance weighted | 41 | 1.08 (1.01- 1.16) | 0.026 | 0.047 |  |
|  | Simple mode | 41 | 1.06 (0.88 - 1.27) | 0.560 |  |  |
|  | Weighted mode | 41 | 1.04 (0.94- 1.16) | 0.432 |  |  |
| **Taurocholate** | |  |  |  |  |  |
|  | MR Egger | 16 | 0.99 (0.97- 1.02) | 0.536 | 0.225 | 0.333 |
|  | Weighted median | 16 | 0.99 (0.97- 1.01) | 0.306 |  |  |
|  | Inverse variance weighted | 16 | 0.98 (0.97- 1.00) | 0.041 | 0.220 |  |
|  | Simple mode | 16 | 0.98(0.95- 1.02) | 0.394 |  |  |
|  | Weighted mode | 16 | 0.98 (0.96- 1.01) | 0.272 |  |  |
| **Dodecanedioate** | |  |  |  |  |  |
|  | MR Egger | 7 | 0.93 (0.76 -1.15) | 0.532 | 0.768 | 0.226 |
|  | Weighted median | 7 | 1.06 (0.99 - 1.13) | 0.098 |  |  |
|  | Inverse variance weighted | 7 | 1.07 (1.01- 1.13) | 0.013 | 0.615 |  |
|  | Simple mode | 7 | 1.06 (0.97 - 1.15) | 0.257 |  |  |
|  | Weighted mode | 7 | 1.05 (0.97 - 1.14) | 0.244 |  |  |
| **12-hydroxyeicosatetraenoate** | |  |  |  |  |  |
|  | MR Egger | 14 | 1.04 (0.97 -1.13) | 0.307 | 0.019 | 0.733 |
|  | Weighted median | 14 | 1.03 (1.01 -1.06) | 0.016 |  |  |
|  | Inverse variance weighted | 14 | 1.03 (1.00 - 1.06) | 0.021 | 0.028 |  |
|  | Simple mode | 14 | 0.99 (0.94 - 1.05) | 0.791 |  |  |
|  | Weighted mode | 14 | 1.03 (0.99 - 1.07) | 0.164 |  |  |
| **5,8-tetradecadienoate** | |  |  |  |  |  |
|  | MR Egger | 13 | 0.99 (0.90 - 1.08) | 0.820 | 0.791 | 0.135 |
|  | Weighted median | 13 | 1.06 (1.00 - 1.12) | 0.055 |  |  |
|  | Inverse variance weighted | 13 | 1.06 (1.02- 1.10) | 0.006 | 0.641 |  |
|  | Simple mode | 13 | 1.07 (0.98 - 1.17) | 0.170 |  |  |
|  | Weighted mode | 13 | 1.06 (0.98 - 1.14) | 0.148 |  |  |
| **Palmitoyl sphingomyelin** | |  |  |  |  |  |
|  | MR Egger | 48 | 1.21 (0.90 -1.63) | 0.209 | 0.026 | 0.456 |
|  | Weighted median | 48 | 1.10 (0.99 -1.22) | 0.068 |  |  |
|  | Inverse variance weighted | 48 | 1.09 (1.00 - 1.18) | 0.042 | 0.028 |  |
|  | Simple mode | 48 | 1.07 (0.82- 1.39) | 0.607 |  |  |
|  | Weighted mode | 48 | 1.09 (0.85 - 1.40) | 0.518 |  |  |
| **15-methylpalmitate** | |  |  |  |  |  |
|  | MR Egger | 14 | 0.89 (0.71- 1.12) | 0.351 | 0.056 | 0.960 |
|  | Weighted median | 14 | 0.94 (0.84 - 1.05) | 0.305 |  |  |
|  | Inverse variance weighted | 14 | 0.89 (0.81 - 0.98) | 0.019 | 0.079 |  |
|  | Simple mode | 14 | 0.98 (0.84 - 1.15) | 0.829 |  |  |
|  | Weighted mode | 14 | 0.96 (0.83- 1.12) | 0.636 |  |  |
| **Lactate** | |  |  |  |  |  |
|  | MR Egger | 12 | 1.05 (0.74 -1.51) | 0.778 | 0.182 | 0.713 |
|  | Weighted median | 12 | 1.11 (0.97- 1.28) | 0.133 |  |  |
|  | Inverse variance weighted | 12 | 1.13 (1.01 - 1.26) | 0.039 | 0.232 |  |
|  | Simple mode | 12 | 1.10 (0.88- 1.39) | 0.418 |  |  |
|  | Weighted mode | 12 | 1.08 (0.89- 1.32) | 0.433 |  |  |
| **Urate** | |  |  |  |  |  |
|  | MR Egger | 22 | 0.61 (0.46 - 0.82) | 0.003 | 6.22e-05 | 0.045 |
|  | Weighted median | 22 | 0.71 (0.62 - 0.81) | 1.34e-06 |  |  |
|  | Inverse variance weighted | 22 | 0.80 (0.68 -0.94) | 0.006 | 1.49e-06 |  |
|  | Simple mode | 22 | 0.82 (0.56 - 1.21) | 0.331 |  |  |
|  | Weighted mode | 22 | 0.70 (0.61- 0.81) | 7.99e-05 |  |  |
| **Pseudouridine** | |  |  |  |  |  |
|  | MR Egger | 27 | 0.93 (0.67 - 1.27) | 0.638 | 0.304 | 0.785 |
|  | Weighted median | 27 | 0.93 (0.79 - 1.09) | 0.375 |  |  |
|  | Inverse variance weighted | 27 | 0.89 (0.79- 0.99) | 0.032 | 0.349 |  |
|  | Simple mode | 27 | 0.97 (0.71 - 1.31) | 0.824 |  |  |
|  | Weighted mode | 27 | 0.95 (0.75- 1.22) | 0.713 |  |  |
| **Phosphate** | |  |  |  |  |  |
|  | MR Egger | 5 | 1.19 (0.97 - 1.46) | 0.203 | 0.719 | 0.708 |
|  | Weighted median | 5 | 1.15 (0.96 - 1.37) | 0.127 |  |  |
|  | Inverse variance weighted | 5 | 1.15 (1.00- 1.32) | 0.049 | 0.825 |  |
|  | Simple mode | 5 | 1.06 (0.85- 1.32) | 0.620 |  |  |
|  | Weighted mode | 5 | 1.17 (0.97 - 1.40) | 0.178 |  |  |
| **Paraxanthine** | |  |  |  |  |  |
|  | MR Egger | 13 | 1.09 (1.00 -1.18) | 0.079 | 0.451 | 0.247 |
|  | Weighted median | 13 | 1.02 (0.98 - 1.07) | 0.272 |  |  |
|  | Inverse variance weighted | 13 | 1.03 (1.01 - 1.06) | 0.019 | 0.414 |  |
|  | Simple mode | 13 | 1.02 (0.95- 1.09) | 0.581 |  |  |
|  | Weighted mode | 13 | 1.02 (0.96- 1.09) | 0.530 |  |  |
| **2-hydroxyacetaminophen sulfate** | |  |  |  |  |  |
|  | MR Egger | 47 | 1.00 (0.99- 1.00) | 0.311 | 0.320 | 0.805 |
|  | Weighted median | 47 | 1.00 (0.99- 1.00) | 0.190 |  |  |
|  | Inverse variance weighted | 47 | 1.00 (1.00- 1.00) | 0.023 | 0.356 |  |
|  | Simple mode | 47 | 1.00 (0.99- 1.00) | 0.598 |  |  |
|  | Weighted mode | 47 | 1.00 (0.99- 1.00) | 0.150 |  |  |
| **4-hydroxyhippurate** | |  |  |  |  |  |
|  | MR Egger | 8 | 0.98 (0.76 - 1.26) | 0.852 | 0.026 | 0.740 |
|  | Weighted median | 8 | 0.95 (0.90 - 1.00) | 0.043 |  |  |
|  | Inverse variance weighted | 8 | 0.93 (0.88 - 0.99) | 0.018 | 0.041 |  |
|  | Simple mode | 8 | 0.95 (0.87 - 1.04) | 0.299 |  |  |
|  | Weighted mode | 8 | 0.95 (0.88 - 1.03) | 0.245 |  |  |
| **Ergothioneine** | |  |  |  |  |  |
|  | MR Egger | 3 | 1.02 (0.88 - 1.19) | 0.806 | 0.657 | 0.818 |
|  | Weighted median | 3 | 1.05 (1.00 - 1.11) | 0.054 |  |  |
|  | Inverse variance weighted | 3 | 1.05 (1.00 - 1.09) | 0.029 | 0.868 |  |
|  | Simple mode | 3 | 1.05 (1.00 -1.11) | 0.198 |  |  |
|  | Weighted mode | 3 | 1.06 (0.99- 1.12) | 0.225 |  |  |
| **X-11795** | |  |  |  |  |  |
|  | MR Egger | 12 | 0.98 (0.79 -1.22) | 0.111 | 0.478 | 0.368 |
|  | Weighted median | 12 | 1.04 (0.94 - 1.14) | 0.049 |  |  |
|  | Inverse variance weighted | 12 | 1.08 (1.00 - 1.16) | 0.038 | 0.489 |  |
|  | Simple mode | 12 | 1.04 (0.88 - 1.22) | 0.083 |  |  |
|  | Weighted mode | 12 | 1.04 (0.89 - 1.21) | 0.078 |  |  |
| **X-12729** | |  |  |  |  |  |
|  | MR Egger | 18 | 0.97 (0.95 - 1.00) | 0.053 | 0.668 | 0.299 |
|  | Weighted median | 18 | 0.98 (0.97- 1.00) | 0.092 |  |  |
|  | Inverse variance weighted | 18 | 0.99 (0.97 - 1.00) | 0.034 | 0.651 |  |
|  | Simple mode | 18 | 0.99 (0.96 - 1.02) | 0.601 |  |  |
|  | Weighted mode | 18 | 0.98 (0.96 - 1.01) | 0.195 |  |  |
| **X-12771** | |  |  |  |  |  |
|  | MR Egger | 16 | 1.00 (0.89- 1.14) | 0.962 | 0.040 | 0.401 |
|  | Weighted median | 16 | 1.04 (0.98- 1.10) | 0.207 |  |  |
|  | Inverse variance weighted | 16 | 1.06 (1.01- 1.11) | 0.028 | 0.041 |  |
|  | Simple mode | 16 | 1.09 (0.99- 1.20) | 0.097 |  |  |
|  | Weighted mode | 16 | 1.05 (0.97 - 1.12) | 0.250 |  |  |
| **X-13477** | |  |  |  |  |  |
|  | MR Egger | 8 | 1.03 (0.81 - 1.30) | 0.819 | 0.449 | 0.381 |
|  | Weighted median | 8 | 1.18 (1.04 - 1.34) | 0.012 |  |  |
|  | Inverse variance weighted | 8 | 1.14 (1.04 - 1.26) | 0.006 | 0.464 |  |
|  | Simple mode | 8 | 1.23 (1.00- 1.50) | 0.087 |  |  |
|  | Weighted mode | 8 | 1.23 (1.01 - 1.49) | 0.078 |  |  |

Abbreviations: SNP, single nucleotide polymorphism; OR, odds ratio; CI, confidence interval.
