# Supplementary material for: Genetic liability to human serum metabolites is causally linked to telomere length: insights from genome-wide Mendelian randomization and metabolic pathways analysis
Source: Front Nutr. 2024 Aug 26;11:1458442. doi: 10.3389/fnut.2024.1458442 (PMC11381963; doi:10.3389/fnut.2024.1458442)
Supplement: Supplementary file 2 [file Data_Sheet_1.ZIP › Supplementary materials/Supplementary Table S4.docx]

**Table S4.** SNPs detailed information of 11 identified metabolites in this MR study.

| Blood metabolites | SNP | Chr | Position | Effect allele | Beta | SE | *P*-value |
| --- | --- | --- | --- | --- | --- | --- | --- |
| taurocholate | rs10514476 | 16 | 80023841 | A | 0.0391 | 0.0082 | 1.803E-06 |
| taurocholate | rs10766562 | 11 | 19505217 | T | 0.0389 | 0.0082 | 0.00000197 |
| taurocholate | rs10813358 | 9 | 30755626 | A | 0.0373 | 0.0082 | 5.616E-06 |
| taurocholate | rs12070179 | 1 | 153742437 | T | -0.2041 | 0.0432 | 2.308E-06 |
| taurocholate | rs12984452 | 19 | 54021597 | T | -0.0413 | 0.009 | 4.705E-06 |
| taurocholate | rs16955093 | 17 | 5692999 | T | 0.3552 | 0.0782 | 5.606E-06 |
| taurocholate | rs17169191 | 7 | 97501547 | A | -0.1825 | 0.0405 | 6.608E-06 |
| taurocholate | rs1948095 | 11 | 98747735 | A | -0.1423 | 0.0286 | 6.394E-07 |
| taurocholate | rs226212 | 17 | 37036554 | A | -0.0384 | 0.0084 | 0.00000484 |
| taurocholate | rs309873 | 4 | 48336820 | C | 0.0743 | 0.0164 | 5.634E-06 |
| taurocholate | rs7108724 | 11 | 129220126 | A | -0.0389 | 0.0082 | 0.00000213 |
| taurocholate | rs7139744 | 13 | 41039186 | T | 0.0423 | 0.0092 | 3.867E-06 |
| taurocholate | rs7171216 | 15 | 98893001 | T | -0.0442 | 0.0096 | 4.406E-06 |
| taurocholate | rs7809441 | 7 | 31155802 | A | 0.0748 | 0.0147 | 3.801E-07 |
| taurocholate | rs9815713 | 3 | 153568131 | A | -0.0917 | 0.0192 | 1.794E-06 |
| taurocholate | rs9962219 | 18 | 61131461 | T | -0.4251 | 0.0954 | 8.279E-06 |
| 15-methylpalmitate | rs10753083 | 1 | 173523580 | T | 0.0107 | 0.0024 | 9.346E-06 |
| 15-methylpalmitate | rs11118895 | 1 | 222093006 | T | 0.021 | 0.0041 | 2.699E-07 |
| 15-methylpalmitate | rs1190713 | 14 | 102005523 | T | 0.031 | 0.007 | 9.486E-06 |
| 15-methylpalmitate | rs12429177 | 13 | 93071017 | T | -0.0657 | 0.0148 | 9.717E-06 |
| 15-methylpalmitate | rs12967799 | 18 | 70116989 | A | 0.0215 | 0.0047 | 4.428E-06 |
| 15-methylpalmitate | rs17155949 | 5 | 103243077 | T | 0.0641 | 0.0142 | 6.587E-06 |
| 15-methylpalmitate | rs1995777 | 5 | 32987956 | T | -0.011 | 0.0024 | 4.659E-06 |
| 15-methylpalmitate | rs3751196 | 12 | 104157502 | A | 0.0221 | 0.0046 | 1.993E-06 |
| 15-methylpalmitate | rs3851552 | 10 | 49799453 | T | 0.0126 | 0.0026 | 1.472E-06 |
| 15-methylpalmitate | rs4765789 | 12 | 4950048 | T | -0.0112 | 0.0024 | 3.883E-06 |
| 15-methylpalmitate | rs6544157 | 2 | 38546918 | A | -0.0186 | 0.0042 | 7.826E-06 |
| 15-methylpalmitate | rs7187181 | 16 | 82274265 | T | 0.0263 | 0.0057 | 0.00000334 |
| 15-methylpalmitate | rs7681360 | 4 | 57973165 | A | 0.0129 | 0.0024 | 1.081E-07 |
| 15-methylpalmitate | rs9809122 | 3 | 70084253 | A | -0.0113 | 0.0024 | 2.926E-06 |
| pseudouridine | rs1051331 | 21 | 46226786 | A | -0.0088 | 0.0018 | 6.47E-07 |
| pseudouridine | rs10809858 | 9 | 12908303 | A | -0.0103 | 0.0022 | 4.158E-06 |
| pseudouridine | rs11047762 | 12 | 25172939 | T | -0.0157 | 0.0033 | 1.489E-06 |
| pseudouridine | rs11617124 | 13 | 83443386 | A | -0.0081 | 0.0018 | 3.783E-06 |
| pseudouridine | rs12041762 | 1 | 88641601 | T | -0.0075 | 0.0017 | 7.845E-06 |
| pseudouridine | rs12197031 | 6 | 139727096 | T | 0.0079 | 0.0018 | 7.226E-06 |
| pseudouridine | rs1287262 | 5 | 36058174 | C | 0.0083 | 0.0017 | 1.158E-06 |
| pseudouridine | rs12906520 | 15 | 70171419 | A | 0.0402 | 0.009 | 0.00000754 |
| pseudouridine | rs13157599 | 5 | 122641073 | T | 0.0171 | 0.0035 | 1.172E-06 |
| pseudouridine | rs17304639 | 19 | 35948797 | A | -0.0094 | 0.0018 | 8.642E-08 |
| pseudouridine | rs17318174 | 10 | 6954242 | A | 0.008 | 0.0017 | 4.203E-06 |
| pseudouridine | rs2209196 | 1 | 209201790 | T | -0.0083 | 0.0017 | 2.213E-06 |
| pseudouridine | rs2465657 | 2 | 201461814 | A | -0.0085 | 0.0017 | 9.903E-07 |
| pseudouridine | rs3853497 | 1 | 108657184 | T | -0.0088 | 0.0017 | 2.908E-07 |
| pseudouridine | rs4129608 | 9 | 18327215 | A | 0.0087 | 0.0018 | 7.162E-07 |
| pseudouridine | rs4697477 | 4 | 24723251 | T | -0.0085 | 0.0018 | 2.191E-06 |
| pseudouridine | rs4707716 | 6 | 92751145 | T | -0.0116 | 0.0026 | 6.072E-06 |
| pseudouridine | rs5749010 | 22 | 17765505 | A | -0.0127 | 0.0028 | 4.424E-06 |
| pseudouridine | rs5761606 | 22 | 26963743 | T | 0.0118 | 0.0026 | 7.858E-06 |
| pseudouridine | rs6434188 | 2 | 187449871 | T | 0.0075 | 0.0017 | 8.961E-06 |
| pseudouridine | rs6543592 | 2 | 31240246 | A | -0.0127 | 0.0026 | 8.841E-07 |
| pseudouridine | rs7090877 | 10 | 130560053 | A | 0.0132 | 0.0026 | 2.563E-07 |
| pseudouridine | rs7939695 | 11 | 2728090 | T | -0.0155 | 0.0033 | 0.00000276 |
| pseudouridine | rs8026775 | 15 | 85511647 | A | -0.0155 | 0.0032 | 1.155E-06 |
| pseudouridine | rs9500927 | 6 | 32962882 | A | 0.0081 | 0.0018 | 6.563E-06 |
| pseudouridine | rs9540840 | 13 | 67184560 | T | 0.0083 | 0.0018 | 3.819E-06 |
| pseudouridine | rs9633478 | 1 | 77885282 | A | -0.0129 | 0.0026 | 5.313E-07 |
| 2-hydroxyacetaminophen sulfate | rs10848757 | 12 | 3084576 | A | -0.9526 | 0.1628 | 4.845E-09 |
| 2-hydroxyacetaminophen sulfate | rs11082714 | 18 | 46389383 | A | 0.3479 | 0.0726 | 1.678E-06 |
| 2-hydroxyacetaminophen sulfate | rs11137198 | 9 | 140638534 | A | -0.7694 | 0.1466 | 1.534E-07 |
| 2-hydroxyacetaminophen sulfate | rs11724451 | 4 | 90771524 | T | -0.682 | 0.1543 | 0.00000985 |
| 2-hydroxyacetaminophen sulfate | rs11778790 | 8 | 78994541 | T | 0.9754 | 0.2162 | 6.453E-06 |
| 2-hydroxyacetaminophen sulfate | rs12426351 | 12 | 106636097 | T | -1.192 | 0.1953 | 1.037E-09 |
| 2-hydroxyacetaminophen sulfate | rs12516510 | 5 | 153979643 | A | 0.1608 | 0.0353 | 5.327E-06 |
| 2-hydroxyacetaminophen sulfate | rs12531802 | 7 | 92680785 | T | -0.6855 | 0.1522 | 6.651E-06 |
| 2-hydroxyacetaminophen sulfate | rs12544572 | 8 | 18671542 | C | -0.3743 | 0.0802 | 3.068E-06 |
| 2-hydroxyacetaminophen sulfate | rs1263280 | 5 | 15204361 | T | -0.783 | 0.1498 | 1.729E-07 |
| 2-hydroxyacetaminophen sulfate | rs12784492 | 10 | 14463299 | A | 0.9912 | 0.1968 | 4.756E-07 |
| 2-hydroxyacetaminophen sulfate | rs12792005 | 11 | 1630177 | A | -0.2024 | 0.0439 | 4.007E-06 |
| 2-hydroxyacetaminophen sulfate | rs12797812 | 11 | 74235336 | T | -0.7626 | 0.1576 | 1.311E-06 |
| 2-hydroxyacetaminophen sulfate | rs12960835 | 18 | 45481926 | T | -1.1492 | 0.1988 | 7.403E-09 |
| 2-hydroxyacetaminophen sulfate | rs13071058 | 3 | 32958723 | A | 0.9598 | 0.2122 | 6.091E-06 |
| 2-hydroxyacetaminophen sulfate | rs162882 | 5 | 131608291 | C | 0.4501 | 0.0874 | 2.569E-07 |
| 2-hydroxyacetaminophen sulfate | rs16892783 | 6 | 39937820 | C | -0.6355 | 0.1216 | 1.731E-07 |
| 2-hydroxyacetaminophen sulfate | rs16964920 | 13 | 105220468 | A | -0.9595 | 0.2128 | 6.537E-06 |
| 2-hydroxyacetaminophen sulfate | rs17022000 | 2 | 38331037 | A | -0.7015 | 0.1313 | 9.157E-08 |
| 2-hydroxyacetaminophen sulfate | rs17061716 | 3 | 59982336 | C | 0.3081 | 0.0679 | 5.619E-06 |
| 2-hydroxyacetaminophen sulfate | rs17095379 | 12 | 45403273 | A | 0.5603 | 0.1119 | 5.451E-07 |
| 2-hydroxyacetaminophen sulfate | rs1806077 | 5 | 9203274 | A | -0.4253 | 0.0889 | 1.728E-06 |
| 2-hydroxyacetaminophen sulfate | rs1911951 | 19 | 31257696 | A | 0.203 | 0.043 | 2.348E-06 |
| 2-hydroxyacetaminophen sulfate | rs1923325 | 10 | 108464633 | T | 0.5416 | 0.1117 | 1.255E-06 |
| 2-hydroxyacetaminophen sulfate | rs265795 | 3 | 1364796 | C | -0.2845 | 0.0613 | 3.508E-06 |
| 2-hydroxyacetaminophen sulfate | rs28409621 | 8 | 37309527 | A | -0.4719 | 0.1019 | 0.00000365 |
| 2-hydroxyacetaminophen sulfate | rs3850788 | 18 | 4413203 | A | -0.3028 | 0.0647 | 2.841E-06 |
| 2-hydroxyacetaminophen sulfate | rs4279445 | 6 | 97698269 | A | -0.9923 | 0.21 | 2.299E-06 |
| 2-hydroxyacetaminophen sulfate | rs4299028 | 13 | 80596188 | A | -0.9695 | 0.2028 | 1.751E-06 |
| 2-hydroxyacetaminophen sulfate | rs4516988 | 6 | 31176602 | A | 0.2638 | 0.0584 | 6.367E-06 |
| 2-hydroxyacetaminophen sulfate | rs4784988 | 16 | 58859968 | T | -0.8149 | 0.1753 | 3.351E-06 |
| 2-hydroxyacetaminophen sulfate | rs4805580 | 19 | 31052575 | T | 0.964 | 0.2118 | 5.351E-06 |
| 2-hydroxyacetaminophen sulfate | rs4951117 | 1 | 204778847 | A | -0.4779 | 0.0752 | 2.047E-10 |
| 2-hydroxyacetaminophen sulfate | rs500237 | 9 | 92809389 | T | -1.2925 | 0.2889 | 7.665E-06 |
| 2-hydroxyacetaminophen sulfate | rs575723 | 6 | 112313190 | A | -0.9929 | 0.2101 | 2.285E-06 |
| 2-hydroxyacetaminophen sulfate | rs6014654 | 20 | 54831880 | A | 0.6332 | 0.1395 | 5.604E-06 |
| 2-hydroxyacetaminophen sulfate | rs6130478 | 20 | 42533655 | A | 0.8585 | 0.1698 | 4.277E-07 |
| 2-hydroxyacetaminophen sulfate | rs6664483 | 1 | 201116722 | T | 1.1464 | 0.1447 | 2.322E-15 |
| 2-hydroxyacetaminophen sulfate | rs690764 | 16 | 84574353 | A | -0.6782 | 0.1147 | 3.355E-09 |
| 2-hydroxyacetaminophen sulfate | rs6912629 | 6 | 148637133 | C | -0.1016 | 0.022 | 3.974E-06 |
| 2-hydroxyacetaminophen sulfate | rs7071224 | 10 | 98318820 | C | 0.3778 | 0.0779 | 1.222E-06 |
| 2-hydroxyacetaminophen sulfate | rs7082110 | 10 | 34161048 | A | 0.1386 | 0.0309 | 7.096E-06 |
| 2-hydroxyacetaminophen sulfate | rs7120657 | 11 | 112589597 | T | -0.6419 | 0.1355 | 2.167E-06 |
| 2-hydroxyacetaminophen sulfate | rs7863822 | 9 | 120455394 | T | -1.0516 | 0.206 | 3.317E-07 |
| 2-hydroxyacetaminophen sulfate | rs787878 | 9 | 135306762 | T | 0.2768 | 0.0609 | 5.512E-06 |
| 2-hydroxyacetaminophen sulfate | rs899311 | 16 | 7092265 | A | 0.4035 | 0.084 | 1.546E-06 |
| 2-hydroxyacetaminophen sulfate | rs9921618 | 16 | 1901721 | T | 0.3006 | 0.068 | 9.923E-06 |
| levulinate | rs10162458 | 14 | 78105188 | T | 0.0087 | 0.0019 | 0.0000085 |
| levulinate | rs10514491 | 16 | 80315263 | T | 0.0088 | 0.002 | 7.285E-06 |
| levulinate | rs109843 | 5 | 98341053 | A | -0.0087 | 0.0019 | 7.145E-06 |
| levulinate | rs11222082 | 11 | 99837258 | C | -0.0137 | 0.0029 | 2.731E-06 |
| levulinate | rs11776902 | 8 | 4170978 | A | -0.0091 | 0.0019 | 3.214E-06 |
| levulinate | rs12406448 | 1 | 10106775 | T | 0.1301 | 0.0292 | 8.127E-06 |
| levulinate | rs12498629 | 4 | 45786807 | T | -0.0086 | 0.002 | 9.338E-06 |
| levulinate | rs12616207 | 2 | 28474423 | A | 0.0099 | 0.002 | 4.164E-07 |
| levulinate | rs12787323 | 11 | 125806187 | T | 0.0093 | 0.002 | 1.646E-06 |
| levulinate | rs12871836 | 13 | 98748212 | A | -0.0093 | 0.0019 | 1.531E-06 |
| levulinate | rs12996267 | 2 | 23296529 | C | -0.0103 | 0.0019 | 1.234E-07 |
| levulinate | rs13268221 | 8 | 27877350 | T | -0.0088 | 0.0019 | 6.217E-06 |
| levulinate | rs13278849 | 8 | 26714874 | A | -0.013 | 0.002 | 2.786E-11 |
| levulinate | rs13395555 | 2 | 85976236 | T | 0.0086 | 0.0019 | 8.249E-06 |
| levulinate | rs1389052 | 3 | 103008371 | T | 0.0087 | 0.002 | 8.954E-06 |
| levulinate | rs1457192 | 8 | 4682675 | A | -0.009 | 0.002 | 6.795E-06 |
| levulinate | rs1630999 | 3 | 10305551 | C | -0.0101 | 0.0019 | 1.93E-07 |
| levulinate | rs17118209 | 14 | 47690236 | T | -0.0291 | 0.0066 | 9.679E-06 |
| levulinate | rs17589032 | 11 | 37683421 | T | -0.0338 | 0.0076 | 8.703E-06 |
| levulinate | rs1769582 | 14 | 34501834 | A | 0.0089 | 0.002 | 5.582E-06 |
| levulinate | rs1876088 | 2 | 51342409 | A | -0.0088 | 0.0019 | 5.768E-06 |
| levulinate | rs1998125 | 14 | 38081005 | T | -0.0088 | 0.0019 | 5.751E-06 |
| levulinate | rs2256490 | 21 | 46087077 | A | 0.0087 | 0.0019 | 7.725E-06 |
| levulinate | rs227375 | 4 | 103618023 | T | 0.0097 | 0.0019 | 5.756E-07 |
| levulinate | rs2286707 | 7 | 23028610 | T | -0.0087 | 0.002 | 9.496E-06 |
| levulinate | rs2434210 | 16 | 10299190 | A | -0.0139 | 0.0029 | 1.301E-06 |
| levulinate | rs2514587 | 8 | 119599692 | A | 0.01 | 0.0019 | 2.999E-07 |
| levulinate | rs2781537 | 9 | 72078593 | T | 0.0087 | 0.0019 | 7.722E-06 |
| levulinate | rs2870925 | 12 | 67313182 | C | -0.0088 | 0.0019 | 5.835E-06 |
| levulinate | rs302149 | 7 | 18286938 | A | 0.0258 | 0.0058 | 9.337E-06 |
| levulinate | rs328370 | 16 | 73797008 | T | 0.0098 | 0.0019 | 5.302E-07 |
| levulinate | rs3742766 | 14 | 76135546 | A | -0.0215 | 0.0048 | 8.441E-06 |
| levulinate | rs3845671 | 2 | 129556690 | C | -0.0087 | 0.0019 | 7.339E-06 |
| levulinate | rs4147331 | 8 | 114991022 | A | 0.0087 | 0.0019 | 7.471E-06 |
| levulinate | rs4298477 | 8 | 40647884 | T | 0.0087 | 0.0019 | 8.401E-06 |
| levulinate | rs4434479 | 6 | 96289803 | A | 0.0096 | 0.0019 | 8.836E-07 |
| levulinate | rs446125 | 20 | 43267265 | T | -0.0128 | 0.0029 | 9.272E-06 |
| levulinate | rs4878628 | 9 | 35766561 | T | 0.0106 | 0.002 | 6.176E-08 |
| levulinate | rs534408 | 11 | 79363044 | T | 0.0088 | 0.0019 | 6.066E-06 |
| levulinate | rs571139 | 11 | 116237017 | A | 0.0093 | 0.0019 | 1.621E-06 |
| levulinate | rs598236 | 19 | 6009445 | T | -0.0092 | 0.0019 | 2.278E-06 |
| levulinate | rs624947 | 11 | 68140782 | A | -0.0094 | 0.002 | 1.898E-06 |
| levulinate | rs625676 | 11 | 131824499 | T | -0.009 | 0.0019 | 3.673E-06 |
| levulinate | rs6441104 | 3 | 156681441 | A | 0.0139 | 0.0029 | 1.725E-06 |
| levulinate | rs6476317 | 9 | 31990046 | T | 0.0186 | 0.0039 | 1.677E-06 |
| levulinate | rs6513545 | 20 | 59472167 | A | 0.0088 | 0.0019 | 5.253E-06 |
| levulinate | rs6738212 | 2 | 224365812 | A | -0.0089 | 0.0019 | 4.294E-06 |
| levulinate | rs6874723 | 5 | 174921170 | T | 0.0091 | 0.0019 | 0.00000317 |
| levulinate | rs6919834 | 6 | 106101469 | T | 0.0144 | 0.0029 | 8.273E-07 |
| levulinate | rs711995 | 3 | 194360927 | T | 0.0108 | 0.0019 | 2.704E-08 |
| levulinate | rs7194277 | 16 | 8571350 | C | -0.0087 | 0.0019 | 8.334E-06 |
| levulinate | rs727233 | 12 | 5080920 | A | -0.0089 | 0.002 | 6.488E-06 |
| levulinate | rs7541235 | 1 | 81765344 | T | -0.0103 | 0.0019 | 1.149E-07 |
| levulinate | rs7677906 | 4 | 178697207 | T | 0.0087 | 0.0019 | 7.071E-06 |
| levulinate | rs7776725 | 7 | 121033121 | T | -0.0086 | 0.0019 | 9.274E-06 |
| levulinate | rs7800932 | 7 | 153620062 | T | 0.0088 | 0.0019 | 5.515E-06 |
| levulinate | rs8180794 | 7 | 125719132 | A | 0.0092 | 0.0019 | 2.017E-06 |
| levulinate | rs892857 | 11 | 78707366 | T | -0.0091 | 0.0019 | 2.833E-06 |
| levulinate | rs922048 | 18 | 56876386 | A | -0.009 | 0.0019 | 3.331E-06 |
| levulinate | rs9539356 | 13 | 62463331 | A | 0.0089 | 0.0019 | 4.746E-06 |
| levulinate | rs9547604 | 13 | 37128625 | A | -0.0087 | 0.002 | 7.715E-06 |
| dodecanedioate | rs11126153 | 2 | 68002545 | A | -0.0205 | 0.0044 | 0.00000322 |
| dodecanedioate | rs1539549 | 13 | 36451881 | T | -0.0369 | 0.0079 | 2.718E-06 |
| dodecanedioate | rs1572603 | 1 | 47272403 | A | -0.036 | 0.0064 | 1.464E-08 |
| dodecanedioate | rs16846919 | 1 | 227265831 | T | -0.0398 | 0.0084 | 2.445E-06 |
| dodecanedioate | rs2903593 | 19 | 31318358 | C | -0.0374 | 0.0081 | 4.521E-06 |
| dodecanedioate | rs602609 | 11 | 85399684 | A | -0.0391 | 0.0088 | 9.276E-06 |
| dodecanedioate | rs7499321 | 16 | 10270314 | T | 0.0192 | 0.0037 | 2.141E-07 |
| 5,8-tetradecadienoate | rs11717259 | 3 | 22106222 | A | -0.0201 | 0.0044 | 4.327E-06 |
| 5,8-tetradecadienoate | rs11883258 | 19 | 31790369 | C | 0.0208 | 0.0043 | 1.049E-06 |
| 5,8-tetradecadienoate | rs12068349 | 1 | 14670045 | A | 0.0556 | 0.0125 | 9.225E-06 |
| 5,8-tetradecadienoate | rs2123517 | 2 | 130197577 | T | 0.1158 | 0.024 | 1.458E-06 |
| 5,8-tetradecadienoate | rs2915420 | 9 | 103955550 | T | -0.0261 | 0.0056 | 3.187E-06 |
| 5,8-tetradecadienoate | rs3770914 | 2 | 36636830 | T | 0.0537 | 0.0116 | 0.00000394 |
| 5,8-tetradecadienoate | rs393228 | 6 | 24962428 | T | 0.034 | 0.0075 | 5.618E-06 |
| 5,8-tetradecadienoate | rs6685187 | 1 | 151858888 | A | -0.0432 | 0.0041 | 2.879E-26 |
| 5,8-tetradecadienoate | rs7621575 | 3 | 176456273 | A | -0.0257 | 0.0058 | 7.757E-06 |
| 5,8-tetradecadienoate | rs7826057 | 8 | 95795266 | A | -0.0345 | 0.0078 | 9.429E-06 |
| 5,8-tetradecadienoate | rs8037595 | 15 | 29665115 | T | -0.0329 | 0.0074 | 9.146E-06 |
| 5,8-tetradecadienoate | rs9552232 | 13 | 21121357 | A | 0.076 | 0.0172 | 9.682E-06 |
| 5,8-tetradecadienoate | rs9654430 | 5 | 75647750 | A | -0.0189 | 0.0042 | 5.924E-06 |
| lactate | rs10788232 | 10 | 123803584 | T | -0.0111 | 0.0024 | 2.464E-06 |
| lactate | rs1193345 | 1 | 17913714 | A | -0.0216 | 0.0045 | 1.434E-06 |
| lactate | rs1330523 | 13 | 104925616 | A | -0.0117 | 0.0024 | 1.561E-06 |
| lactate | rs13403441 | 2 | 83225802 | A | -0.0235 | 0.0048 | 1.019E-06 |
| lactate | rs1876100 | 9 | 81189702 | A | -0.0106 | 0.0024 | 9.204E-06 |
| lactate | rs2000812 | 18 | 47093790 | T | 0.0111 | 0.0025 | 0.0000097 |
| lactate | rs2242578 | 12 | 57853153 | C | -0.0111 | 0.0024 | 5.078E-06 |
| lactate | rs3785024 | 16 | 84136233 | A | -0.0223 | 0.0049 | 5.562E-06 |
| lactate | rs4635489 | 2 | 30126856 | A | 0.0364 | 0.008 | 5.663E-06 |
| lactate | rs4718532 | 7 | 66805000 | T | -0.016 | 0.0035 | 6.605E-06 |
| lactate | rs723526 | 7 | 55139751 | A | -0.011 | 0.0024 | 6.624E-06 |
| lactate | rs780093 | 2 | 27742603 | T | 0.0117 | 0.0023 | 1.887E-07 |
| phosphate | rs10404980 | 19 | 46758941 | A | -0.0744 | 0.0158 | 2.337E-06 |
| phosphate | rs1466522 | 8 | 85520966 | T | -0.0067 | 0.0013 | 3.149E-07 |
| phosphate | rs2921879 | 2 | 114331775 | T | -0.0055 | 0.0012 | 8.665E-06 |
| phosphate | rs4641759 | 16 | 62531768 | T | -0.0135 | 0.003 | 0.00000666 |
| phosphate | rs6126354 | 20 | 50470585 | T | -0.0134 | 0.0029 | 5.463E-06 |
| paraxanthine | rs10791723 | 11 | 104554703 | A | -0.0658 | 0.0141 | 2.962E-06 |
| paraxanthine | rs11915650 | 3 | 174942871 | A | -0.0474 | 0.01 | 2.221E-06 |
| paraxanthine | rs12341812 | 9 | 17107467 | T | -0.093 | 0.0188 | 7.435E-07 |
| paraxanthine | rs12895893 | 14 | 26708629 | A | -0.0443 | 0.0093 | 1.964E-06 |
| paraxanthine | rs13387835 | 2 | 107142307 | A | -0.0622 | 0.0138 | 6.237E-06 |
| paraxanthine | rs17777937 | 8 | 84937766 | T | -0.0744 | 0.0165 | 6.198E-06 |
| paraxanthine | rs2059238 | 16 | 78258810 | A | -0.0435 | 0.0087 | 6.602E-07 |
| paraxanthine | rs29456 | 5 | 65539847 | T | -0.0356 | 0.0077 | 4.097E-06 |
| paraxanthine | rs3768372 | 1 | 226835632 | C | -0.0808 | 0.0182 | 8.723E-06 |
| paraxanthine | rs3846408 | 4 | 14132506 | C | 0.0303 | 0.0068 | 9.478E-06 |
| paraxanthine | rs4661452 | 1 | 14272107 | T | -0.0577 | 0.0127 | 5.176E-06 |
| paraxanthine | rs7558224 | 2 | 239847530 | T | 0.0376 | 0.0084 | 8.564E-06 |
| paraxanthine | rs946712 | 9 | 134389070 | A | 0.084 | 0.0165 | 3.294E-07 |
| ergothioneine | rs12727717 | 1 | 18393640 | T | -0.1065 | 0.0226 | 2.374E-06 |
| ergothioneine | rs4823691 | 22 | 48178666 | T | -0.1683 | 0.0378 | 8.665E-06 |
| ergothioneine | rs6082720 | 20 | 2302087 | T | 0.2245 | 0.05 | 7.028E-06 |

Abbreviations: Chr, chromosome; SE, standard error; SNP, single nucleotide polymorphism.
