# Supplementary material for: Genetic liability to human serum metabolites is causally linked to telomere length: insights from genome-wide Mendelian randomization and metabolic pathways analysis
Source: Front Nutr. 2024 Aug 26;11:1458442. doi: 10.3389/fnut.2024.1458442 (PMC11381963; doi:10.3389/fnut.2024.1458442)
Supplement: Supplementary file 2 [file Data_Sheet_1.ZIP › Supplementary materials/Supplementary Table S5.docx]

**Table S5.** Significant metabolic pathways involved in the biological process of telomeres development.

| **Trait** | **Metabolic pathway** | **Metabolites involved** | **P-value** | **Database** |
| --- | --- | --- | --- | --- |
| Telomere length | Taurine and hypotaurine metabolism | Taurocholate | 0.031 | KEGG |
| Telomere length | Caffeine metabolism | 1,7-Dimethylxanthine | 0.038 | KEGG |
